# Leveraging Public Single-Cell and Bulk Transcriptomic Datasets to Delineate MAIT Cell Roles and Phenotypic Characteristics in Human Malignancies

**DOI:** 10.3389/fimmu.2020.01691

**Published:** 2020-07-31

**Authors:** Tony Yao, Parisa Shooshtari, S. M. Mansour Haeryfar

**Affiliations:** ^1^Department of Microbiology and Immunology, Western University, London, ON, Canada; ^2^Department of Pathology and Laboratory Medicine, Western University, London, ON, Canada; ^3^Lawson Health Research Institute, London Health Sciences Centre, London, ON, Canada; ^4^Division of Clinical Immunology and Allergy, Department of Medicine, Western University, London, ON, Canada; ^5^Division of General Surgery, Department of Surgery, Western University, London, ON, Canada; ^6^Centre for Human Immunology, Western University, London, ON, Canada

**Keywords:** MAIT cells, cancer, transcriptomics, RNA sequencing, TCGA, survival, PD-1, checkpoint inhibitors

## Abstract

Mucosa-associated invariant T (MAIT) cells are unconventional, innate-like T lymphocytes that recognize vitamin B metabolites of microbial origin among other antigens displayed by the monomorphic molecule MHC class I-related protein 1 (MR1). Abundant in human tissues, reactive to local inflammatory cues, and endowed with immunomodulatory and cytolytic functions, MAIT cells are likely to play key roles in human malignancies. They accumulate in various tumor microenvironments (TMEs) where they often lose some of their functional capacities. However, the potential roles of MAIT cells in anticancer immunity or cancer progression and their significance in shaping clinical outcomes remain largely unknown. In this study, we analyzed publicly available bulk and single-cell tumor transcriptomic datasets to investigate the tissue distribution, phenotype, and prognostic significance of MAIT cells across several human cancers. We found that expanded MAIT cell clonotypes were often shared between the blood, tumor tissue and adjacent healthy tissue of patients with colorectal, hepatocellular, and non-small cell lung carcinomas. Gene expression comparisons between tumor-infiltrating and healthy tissue MAIT cells revealed the presence of activation and/or exhaustion programs within the TMEs of primary hepatocellular and colorectal carcinomas. Interestingly, in basal and squamous cell carcinomas of the skin, programmed cell death-1 (PD-1) blockade upregulated the expression of several effector genes in tumor-infiltrating MAIT cells. We derived a signature comprising stable and specific MAIT cell gene markers across several tissue compartments and cancer types. By applying this signature to estimate MAIT cell abundance in pan-cancer gene expression data, we demonstrate that a heavier intratumoral MAIT cell presence is positively correlated with a favorable prognosis in esophageal carcinoma but predicts poor overall survival in colorectal and squamous cell lung carcinomas. Finally, in colorectal carcinoma and four other cancer types, we found a positive correlation between *MR1* expression and estimated MAIT cell abundance. Collectively, our findings indicate that MAIT cells serve important but diverse roles in human cancers. Our work provides useful models and resources that employ gene expression data platforms to enable future studies in the realm of MAIT cell biology.

## Introduction

The composition and the activity of immune cells within tumor microenvironments (TMEs) are important determinants of cancer progression or anticancer immunity and ultimately of clinical outcomes (1, 2). T cells in particular play key roles in host responses to cancer, and their ability to infiltrate tumors is usually associated with a favorable prognosis in various malignancies (3, 4). Conventional CD8^+^ T cells are capable of recognizing and killing tumor cells that display peptide antigens in the context of major histocompatibility complex (MHC) class I molecules. Many cancer immunotherapies in development or in clinical use aim to harness the antitumor functions of neoantigen-specific T cells. Although such and similar strategies have yielded remarkable responses at least in some patients, their broad applicability is curtailed by the polymorphic (i.e., patient-restricted) nature of MHC molecules and by the scarcity of tumor-reactive naïve T cells present in one's T cell repertoire (5–8).

A growing body of evidence has implicated unconventional T cells, including γδ T cells (9–11), invariant natural killer T (*i*NKT) cells (12–15), and more recently mucosa-associated invariant T (MAIT) cells (16–26), in human malignancies. MAIT cells are evolutionarily conserved, innate-like T cells that recognize riboflavin precursor derivatives of microbial origin presented by MHC class I-related protein 1 (MR1) (27–29). The T cell receptor (TCR) of human MAIT cells is composed of a semi-invariant TCRα chain (TRAV1-2–TRAJ33/12/20) that pairs with members of a biased TCRβ repertoire (predominantly TRBV6 and TRBV20) (30–32). Several features of MAIT cells make them likely participants in either protective or pathogenic host responses to cancer and attractive targets in immunotherapeutic strategies. First is their localization at common sites of oncogenesis, tumor growth and metastasis. MAIT cells are abundant in humans, comprising ~1–10% of all T cells in the peripheral blood, ~5–30% in the liver, and also residing in the gut, lungs, kidneys, skin, breasts, and the female genital tract (33–41). Second, MAIT cell activation is readily triggered by inflammatory cytokines such as interleukin (IL)-12 and IL-18 independently of TCR signaling, and can be further tuned by IL-7, IL-15, IL-23, and type I interferons (IFNs) (42–45). Activated MAIT cells can produce a wide array of immunomodulatory cytokines, including IFN-γ, tumor necrosis factor (TNF)-α, and IL-17A, with varying secretion profiles depending on the tissue contexts and the means and modes of MAIT cell stimulation (30, 33, 34). Each of these cytokines can potentially influence cancer progression or antitumor defense mechanisms (46, 47). Lastly, upon encounter with MR1^+^ target cells, MAIT cells degranulate to release perforin and granzymes (48, 49). MAIT cell-mediated oncolysis has yet to be demonstrated *in vivo*. However, MAIT cells were shown to destroy myeloma cell lines pulsed with MR1 ligands *in vitro* (22).

Several clinical studies have demonstrated MAIT cell infiltration into kidney and brain tumors (24), colorectal carcinoma (CRC) and their liver metastases (16–18, 25, 26), multiple myeloma (22), hepatocellular carcinoma (HCC) (19, 20), and esophageal adenocarcinoma (EAC) (21). Declines in circulating MAIT cell frequencies are observed in patients with gastric, lung, liver and colorectal cancers, potentially reflecting MAIT cell recruitment to tumor sites (16, 25). The extent of intratumoral MAIT cell accumulation appears to vary by cancer type. For example, they are enriched in CRC and EAC tumors relative to surrounding normal tissues. In contrast, they can be scarce within HCC tumors and colorectal liver metastases (16–20, 26). Upon *ex vivo* stimulation, MAIT cells isolated from tumors often exhibit impaired T helper-type 1 (T_H_1) functionality, and in certain cancers shift toward a T_H_17 cytokine profile (16, 18, 20–22, 26). A negative correlation between tumor infiltration by MAIT cells and patient survival was reported in CRC, while studies on HCC have yielded contradictory prognostic associations (17, 19, 20). A recent study by Yan et al. addressed the *in vivo* significance of MAIT cells in cancer immunity (50). These investigators found MAIT cells to be immunosuppressive and to promote tumor progression in mouse models of lung metastasis and fibrosarcoma. Of note, mouse and human MAIT cells differ in certain characteristics, for instance in terms of their bias toward a T_H_17 program (51).

The rational design of treatments that target MAIT cells in cancer will first require thorough characterization of their effector mechanisms and roles across various malignancies. MAIT cell-based therapies may offer unique therapeutic advantages over those focusing on conventional T cells. Since MAIT cell ligands are presented in the context of the same antigen-presenting molecule (i.e., MR1) uniformly expressed in all individuals, cognate MAIT cell activation strategies will not be restricted by inter-patient differences dictated by MHC polymorphism (27). Moreover, the high expression level of multi-drug resistance protein 1 (MDR1) by MAIT cells enables them to excrete intracellular toxins, which in turn confers upon them resistance to certain chemotherapies (33). Therefore, combination therapies with MR1-restricted ligands and chemotherapeutic agents should be possible.

Recent advances in sequencing technologies have enabled the generation of large-scale transcriptomic datasets that represent global profiles of TMEs (52–55). A number of methods have been developed to infer from these datasets the relative abundances of different cell populations within tumor samples (56–58). Application of these techniques to The Cancer Genome Atlas (TCGA), a consortium that provides open access to molecular and clinical data across a wide range of cancer types, has facilitated pan-cancer analyses of tumor-infiltrating immune cell subsets (59, 60). Interestingly, one such study identified *KLRB1*, which encodes CD161, a cell surface protein that is highly expressed on MAIT cells, as the most favorable prognostic gene across 39 human malignancies (61). MAIT cells, however, have yet to be studied through this lens. Therefore, in this study, we leveraged available single-cell and bulk tumor transcriptomic datasets to characterize the abundance, clonality and transcriptional profiles of tumor-infiltrating MAIT cells. These analyses also provided us with a unique opportunity to test the impact of immunotherapy with anti-programmed cell death-1 (PD-1) in skin cancer-infiltrating MAIT cells. Moreover, we defined a general MAIT cell gene signature whose association with clinical outcomes we explored in a wide range of human cancers. We validate and extend some of the previous findings on MAIT cell activation and exhaustion within tumors. Our findings uncover several associations, both poor and favorable, between the MAIT cell signature and patient survival. Our work also supplies evidence in support of MAIT cells' participation in host responses to cancer and highlights potential links between MAIT cells and the progression of certain tumors, which warrant further investigation.

## Materials and Methods

### Analysis of Single-Cell RNA Sequencing (scRNA-seq) Datasets Generated by Zheng et al. (19), Zhang et al. (62), and Guo et al. (63)

Pre-processed data from three scRNA-seq studies on human HCC (19), CRC (62), and non-small cell lung cancer (NSCLC) (63), were downloaded from the Gene Expression Omnibus (GEO accessions GSE98638, GSE108989, and GSE99254, respectively) (Supplementary Table 1). Each of these datasets contains gene expression profiles and assembled TCR sequences of single T cells isolated from the peripheral blood, tumor masses, and tumor-adjacent normal tissues of cancer patients. Transcriptomic data from these projects were made available in the form of raw read counts, transcripts per million (TPM), and normalized-centered expression. Different forms of data were used as input depending on the mode of analysis. We analyzed only cells in which at least one productive TCRα-β pair was identified according to the results of the original articles. T cell clonotypes were defined as harboring unique TCRα-β pairs, and cells were considered clonal if they contained the same clonotype. MAIT cells were subsequently defined as those bearing the canonical TRAV1-2-TRAJ33/12/20 semi-invariant TCRα chain. For the remaining cells, annotated identities were retrieved from the original articles and re-classified into more broadly defined and less microenvironment-specific subsets [i.e., CD8^+^ T cells, CD4^+^ T cells, and regulatory T (Treg) cells]. The relative tissue preference of MAIT cells was quantified as *R*_O/E_, the ratio of observed to expected cell number from chi-square tests.

Raw read count data were used as input to generate *t*-distributed stochastic neighbor embedding (*t*-SNE) plots with the Seurat pipeline (64). Matrices were transformed by the LogNormalize function, and highly variable features were detected by the “vst” method of FindVariableFeatures (0.0125 < mean < 8, dispersion >0.5). After applying these steps to the dataset for each cancer, data were combined using the FindIntegrationAnchor and IntegrateData functions (default value of 30 dimensions). Scaled data from the integrated matrix were used for principal component analysis (PCA), from which the top 15 principal components were used for *t*-SNE.

Normalized expression data were used as input for identifying differentially expressed genes (DEGs) and MAIT cell marker genes. The limma-trend procedure was used to identify DEGs between MAIT cells within different tissue compartments. Given the power constraint imparted by the relatively low number of normal tissue MAIT cells sampled in CRC and NSCLC, a hierarchical approach was taken wherein the limma-trend F-statistic was used to first prioritize genes showing signals of differential expression among groups (Benjamini-Hochberg adjusted *p* < 0.2) before conducting pairwise comparisons between compartments (65). Cut-offs for significant DEGs were two-sided unpaired Benjamini-Hochberg adjusted *p* < 0.05 and fold-change ≥1.5. Results from this analysis can be found in Supplementary Table 2. Pathway enrichment analysis was conducted using the GSVA package (66) with the “Gene Ontology (GO) biological process” collection of gene sets from the Molecular Signatures Database (MSigDB) (67). Differentially enriched gene sets in MAIT cells from different tissue compartments were identified with limma-trend following a similar hierarchical approach. For activation and exhaustion scores, log_2_ (TPM+1) of the constituent genes was computed, z-score-transformed, averaged, and then plotted across all cells. The genes utilized to generate the activation scores were *CD69, CD38, HLA-DRA, IL2RA, TNFRSF9, TNF, GNLY, IFNG, GZMB, GZMA*, and *GZMH*. The genes included in the exhaustion scores were *PDCD1, CTLA4, TIGIT, CXCL13, ENTPD1, LAG3, ITGAE*, and *LAYN*. In addition, we tested the 28-gene activation-independent exhaustion program derived by Tirosh et al. (68) as validation for our exhaustion signature.

A consensus-based method was employed to define MAIT signature genes that were stable across tissue compartments as well as cancer types. For each compartment of each cancer dataset, genes exhibiting higher expression in MAIT cells relative to each of the other subsets were identified by limma-trend (two-sided unpaired Benjamini-Hochberg adjusted *p* < 0.1 and fold-change ≥1.5). The intersection of these nine sets of DEGs (three tissues × three cancers), listed in Supplementary Table 3, were designated as MAIT signature genes for downstream analyses. Given the low number of normal tissue MAIT cells (*n* = 15) sampled in the CRC dataset, these were jointly analyzed with intratumoral MAIT cells in the DEG identification step. MAIT signature scores were computed on a per-cell basis in a similar fashion as activation and exhaustion scores [i.e., z-scoring before averaging log_2_ (TPM+1) values].

### Analysis of Datasets Generated by Gutierrez-Arcelus et al. (69)

Gene expression data of innate-like T cells from the peripheral blood of healthy donors were obtained from GSE124731 (69). For bulk RNA sequencing of sorted lymphocyte populations, normalized log_2_ (TPM+1) values were used in downstream analyses. To compare the expression of MAIT signature genes between cell subsets, we averaged data from technical replicates and used the resulting matrix as input for limma-trend, including donor as a covariate. Reported *p* values from this step were left unadjusted as we were testing for specific gene-wise, rather than genome-wide, differential expression. The MAIT signature was scored in each sample by averaging log_2_ (TPM+1) values of the eleven genes, and then compared between cell types by the Wilcoxon signed-rank test. For the scRNA-seq dataset, we used the normalized and log-transformed unique molecular identifier (UMI) count matrix. Gene expression was scaled as z-scores and visualized in a heatmap. MAIT signature scores were computed and compared in the same manner as for the bulk RNA-seq data.

### Analysis of Datasets Generated by Ma et al. (70)

Single-cell transcriptomic results of primary liver tumors were obtained from GSE125449 (70). We jointly analyzed data from HCC patients in both discovery and validation cohorts, excluding those with intrahepatic cholangiocarcinoma. In addition to the quality control measure implemented in the original publication (i.e., excluding cells in which fewer than 500 genes were detected), we filtered out cells with <700 or >25,000 UMI counts, >4,000 genes, or >20% mitochondrial counts. Datasets from the two cohorts were harmonized according to the standard Seurat v3 integration workflow (71). The LogNormalize and FindVariableFeatures (top 2,000 genes by the “vst” method) functions were independently applied to each matrix before FindIntegrationAnchors and IntegrateData (30 dimensions) were called to combine the matrices. During this step, MAIT signature genes as well as cell lineage-specific markers reported in the paper were appended to the list of features to integrate, thus facilitating downstream comparisons. After scaling the combined matrix and performing PCA, the top 20 principal components were used for *t*-SNE. Cell type identities were assigned as those listed in the accompanying metadata files, and unclassified cells were excluded from the *t*-SNE projection. The AddModuleScore function (100 control features, 20 bins) was used to score each cell for the MAIT signature. A correlation matrix of MAIT signature genes and pan-T cell markers (*CD3D, CD3E* and *CD3G*) was generated using log-normalized expression values by computing pairwise Kendall's τ coefficients, followed by hierarchical clustering by centroid linkage to order the genes.

### Analysis of Datasets Generated by Yost et al. (72)

scRNA-seq datasets profiling T cells from site-matched basal and squamous cell carcinoma (BCC and SCC) samples, before and after treatment with PD-1-based checkpoint inhibitors, were retrieved from GSE123814 (72). In addition to UMI count matrices, cell-level metadata and TCR sequences, we obtained from the authors additional information pertaining to V, D and J gene segment usage. As with the other cancer scRNA-seq datasets, we re-classified T cell subsets into CD8^+^, CD4^+^, Treg, and MAIT cells, with the latter being defined based on their expression of TRAV1-2 and TRAJ33/12/20. T cells were marked as clonal if they shared at least one productive TCRα and β nucleotide sequence each. To ensure consistency in our analyses, we re-processed the UMI count data using a workflow similar to the ones we applied to HCC, CRC, and NSCLC datasets (19, 62, 63). However, certain parameters were modified given the difference in sequencing platforms (i.e., 10X droplet-based vs. Smart-seq2 full-length protocol). For quality control, cells were excluded if they had <1,200 or >25,000 UMI molecules, <600 or >4,000 unique detected genes, or >10% mitochondrial counts. Genes were filtered if their average count across all cells was <0.01. Hierarchical clustering of cells based on Spearman's rank correlation was performed with the quickCluster function of the scran package, and size factors were deconvolved within each cluster using the pooling-based computeSumFactors method (73), and gene expression was accordingly log-normalized. Finally, the expression of each gene in each patient was scaled such that the mean was equal to 0. The resulting normalized-centered matrix was used for differential expression analysis using limma-trend, with the fold-change cut-off for significant DEGs set at 1.25.

*t*-SNE plots were generated with the Seurat package using raw UMI counts as the input. For both BCC and SCC, we followed the standard integration workflow to harmonize data from different patients, as cells otherwise clustered by donor, indicative of batch effects. We split the dataset for each cancer by patient, log-normalized and identified highly variable genes (top 2,000 by “vst” method) in these matrices separately, and subsequently called FindIntegrationAnchors and IntegrateData (30 dimensions) to re-join the data. In line with the original article, TCR and immunoglobulin V gene segments were excluded from variable features. A second round of integration enabled us to combine the two cancer datasets, after which CellCycleScoring was used to compute S and G2/M phase scores in each cell using published gene sets (68), and AddModuleScore was used to measure the heat shock response based on the gene ontology term “response to heat.” Gene expression was then scaled and regressed by the number of UMI molecules as well as the S, G2/M, and heat shock scores. Finally, PCA was performed, and RunTSNE was called using the top 20 principal components. Cells without known TCR sequences were excluded from the *t*-SNE projection since they cannot be definitively identified as MAIT or non-MAIT cells.

### Pseudo-Bulk Sample Simulation

Single-cell gene expression data from Yost et al. (72) were used to generate pseudo-bulk RNA-seq samples for validation of the MAIT signature among T cells infiltrating BCC and SCC tumors. We first scored the MAIT signature in each cell by z-scoring the normalized expression of the constituent genes and taking their mean. Certain MAIT signature genes were pre-filtered from the normalized expression matrices owing to their low expression (*ME1, COLQ, ZBTB16*, and *TLE1* for BCC; *SLC4A10, ME1, IL23R, COLQ*, and *ZBTB16* for SCC). This step ensured that the MAIT signature was enriched in MAIT cells from both BCC and SCC tumors before and after anti-PD-1 therapy.

To generate pseudo-bulk samples, we randomly sampled *n* cells from the single-cell dataset without replacement and then summed up the raw UMI counts for each gene across the *n* cells. Since we knew the cell type identities of the sampled cells, we could determine the frequency of MAIT cells in every pseudo-bulk sample as a ground-truth measure. In each artificial sample, we divided the count of every gene by the library size (i.e., total number of UMI counts), multiplied by 10^6^, added one, then took the log_2_ value to produce an expression measure analogous to log_2_ (TPM+1). Next, we averaged the normalized expression of the 11 signature genes to derive the MAIT score. To evaluate the performance of the signature, we calculated Pearson's correlation coefficient and associated *p* values to assess the association between MAIT scores and MAIT cell frequencies across all simulated samples. Outlier MAIT scores, defined as those falling outside three median absolute deviations, were removed before evaluating the correlation. In the first round of simulations, we set *n* as a random integer between 200 and 5,000 and generated 500 samples from the BCC dataset, containing both pre- and post-treatment samples. In the second round, we set *n* to 3,000 and generated 300 samples from each of the BCC and SCC datasets, once for pre-treatment samples and once for post-treatment samples.

### TCGA Analysis

Bulk transcriptomic data from the TCGA Pan-Cancer dataset (TOIL RSEM tpm) were downloaded from UCSC Xena (http://xena.ucsc.edu/) and re-computed as log_2_ (TPM+1) (74). Matching clinical data for 20 TCGA cancers (Supplementary Table 1) were retrieved from cBioPortal (https://www.cbioportal.org/) (59).

In order to validate the MAIT signature derived from scRNA-seq data, a gene expression correlation matrix was generated for each cancer to evaluate the strength of association between MAIT cell (*SLC4A10, KLRB1, IL23R, NCR3, TMIGD2, LST1, COLQ, ME1, ZBTB16, RORC, TLE1*), pan-T cell (*CD3D, CD3E, CD3G*) and NK cell (*XCL2, PRF1, KLRF1, KLRD1, IL2RB, CD244, CD160*) gene markers. The non-parametric and tie-robust Kendall's rank correlation coefficient (Kendall's τ) was used to determine association.

For each cancer, the set of genes comprising the finalized MAIT signature was separately defined according to the following *ad hoc* rules. Of the eleven genes in the full signature, we retained only those that (1) could be significantly correlated with *SLC4A10*, which is consistently the most specific MAIT cell-expressed gene across transcriptomic datasets (19, 62, 63, 75, 76), (2) were correlated with all other genes or all but one gene comprising the finalized MAIT signature, and (3) were correlated with at least two pan-T cell markers. This step was implemented to specifically discard MAIT signature genes whose expression appeared uncoupled with other MAIT and T cell markers, possibly owing to their expression by non-T cells, and which could thus otherwise confound the estimation of MAIT cell abundance if retained. After applying these conditions, only cancers in which at least five genes remained in the MAIT cell signature were analyzed downstream.

In each tumor sample, the MAIT cell score was computed by z-score-transforming and averaging the log_2_(TPM+1) of all constituent genes in the respective MAIT signature for that cancer type. The T cell score in each sample was similarly calculated by using *CD3D, CD3E* and *CD3G*. Before conducting survival analyses, MAIT cell scores were normalized by performing regression on T cell scores and keeping the residuals.

### Survival Analysis

Across 16 cancers in the TCGA, univariate cox proportional hazard models were tested with the MAIT signature score as a predictor for either overall survival (OS) or progression-free survival (PFS). Patients were stratified by MAIT scores at 33rd and 67th percentiles. Significant associations identified in these models were visualized by generating Kaplan-Meier curves.

### Software

Analyses and most visualizations were performed using R (v3.6.1). The packages limma, Seurat, scran, biomaRt, survival, dplyr, and tidyverse were used to manipulate, format and analyze transcriptomic and clinical data. The packages ggplot2, ggsci, ggExtra, ggfortify, ggcorrplot, RColorBrewer, gridExtra, and survminer were used for visualization. The web tool Intervene (https://asntech.shinyapps.io/intervene/) was used to generate UpSet plots that visualized in each cancer scRNA-seq dataset the intersection of MAIT cell markers between blood, normal tissue and tumors. For validation of MAIT signature genes in the Human Blood Atlas, images were obtained directly from the web interface https://www.proteinatlas.org/humanproteome/blood (77).

## Results

### Tissue Distribution of MAIT Cell Clonotypes in Cancer

**Figure 1:**
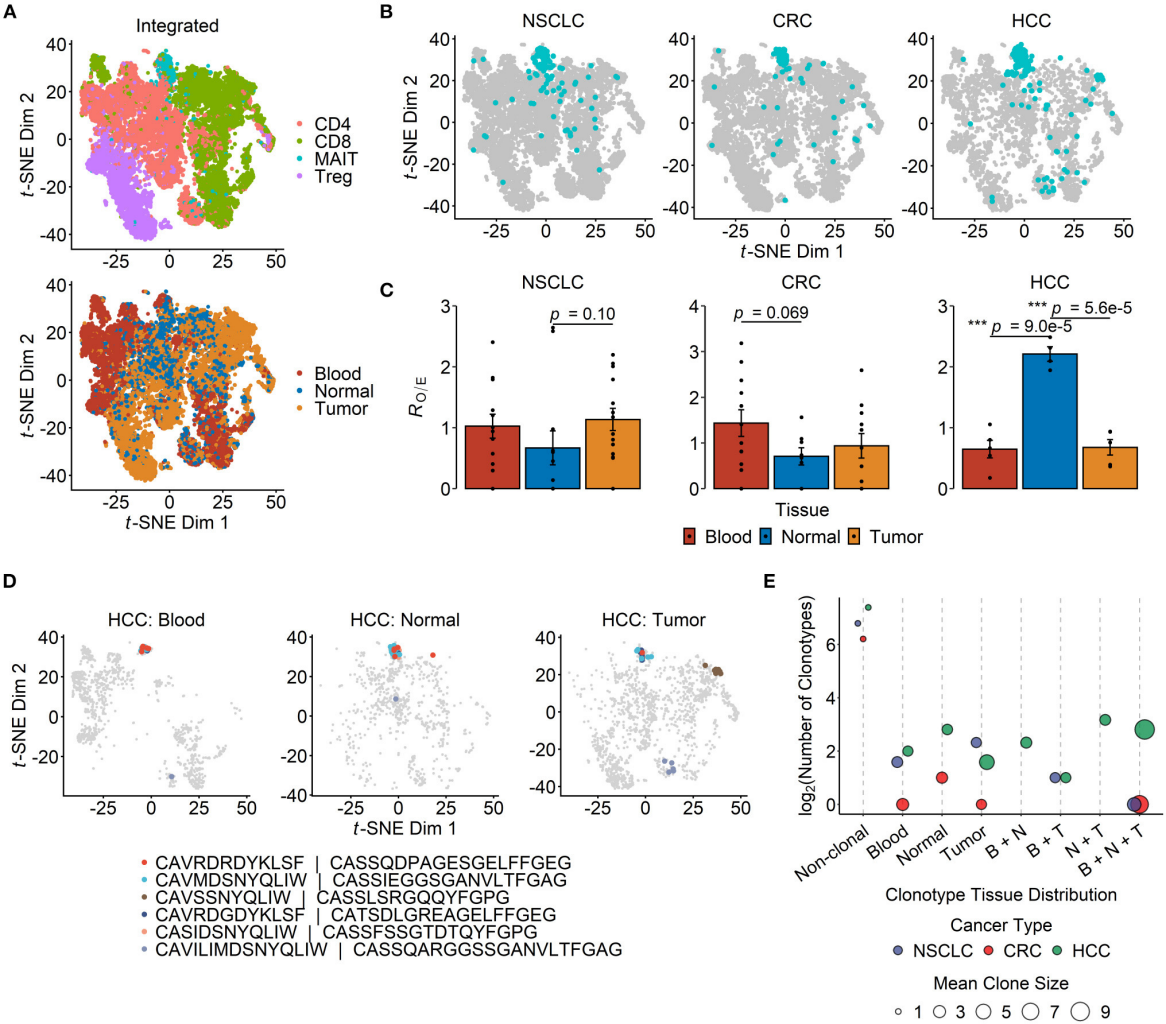
Tissue distribution and clonotype sharing of MAIT cells in cancer. **(A)** *t*-distributed stochastic neighbor embedding (*t*-SNE) projection of 19,435 T cells from integrated non-small cell lung cancer (NSCLC) (*n* = 8,092), colorectal cancer (CRC) (*n* = 7,903) and hepatocellular carcinoma (HCC) (*n* = 3,440) single-cell transcriptomic datasets. Dots depict individual cells, which are colored according either to the T cell subset they are assigned to (top panel) or to their site of origin (bottom panel). **(B)** T cells from each of the three cancer types in **(A)** are plotted separately, with MAIT cells highlighted in blue. **(C)** Relative abundance of MAIT cells across tissue compartments. Tissue preference is quantified as *R*_O/E_, the ratio of observed to expected cell frequency in a chi-squared test. Each dot represents a patient (*n* = 14 for NSCLC, *n* = 12 for CRC, *n* = 5 for HCC), and error bars represent standard error of the mean (SEM). ***p < 0.001; two-sided unpaired student's *t*-test. **(D)** *t*-SNE plots visualizing T cells in the peripheral blood (*n* = 1,063), normal liver tissue (*n* = 819) and tumor tissue (*n* = 1,558) of HCC patients, with the six most abundant MAIT cell clonotypes highlighted in different colors. The complementarity determining region (CDR)3α and CDR3β amino acid sequences are listed for each depicted clonotype. **(E)** Sharing of MAIT cell clonotypes between tissue compartments. The number of unique clonotypes is shown for non-clonal MAIT cells, and so are the tissue combinations of clonal MAIT cell distribution across the three cancer types. Dot sizes indicate the mean number of cells per clonotype group. B, N and T stand for blood, normal tissue and tumor, respectively.

The application of scRNA-seq in cancer immunology has enabled deep, comprehensive and unbiased profiling of immune cell populations within TMEs. We performed a secondary analysis of published scRNA-seq datasets across three human cancers, namely HCC, CRC and NSCLC (19, 62, 63), with a focus on the abundance and the transcriptional profiles of MAIT cells from sampled tissues (Supplementary Table 1). The studies conducted by Zhang's group generated full-length TCR sequencing data for single T cells isolated from the peripheral blood, tumor and paired adjacent normal tissues of cancer patients. MAIT cells, defined based on the expression of their semi-invariant TCRα chain (TRAV1-2-TRAJ33/12/20), were detectable in all three cancer types and formed a distinct cluster when visualized on a *t*-SNE projection (Figures 1A,B). In CRC, there was a trend toward MAIT cell enrichment in blood compared with the normal colonic tissue (*p* = 0.069), but not compared with the tumor tissue (Figure 1C). In contrast, we found MAIT cells to be more abundant in the normal liver tissue than in the tumors or blood circulation of HCC patients (Figure 1C).

Clonal MAIT cells — that is MAIT cells bearing identical TCRα-β pairs — could be detected in all three cancers, and some clonotypes were found in multiple compartments. Of the six most abundant MAIT cell clonotypes in HCC, five were shared between blood, healthy liver and tumor tissues (Figure 1D). Across the three cancer types, the most frequent MAIT clonotypes tended to be shared across all tissue compartments, while the majority of MAIT cells were non-clonal (Figure 1E). These findings are consistent with the notion that MAIT cells may be recruited from the circulation or surrounding tissues into inflamed TMEs.

**Figure 2:**
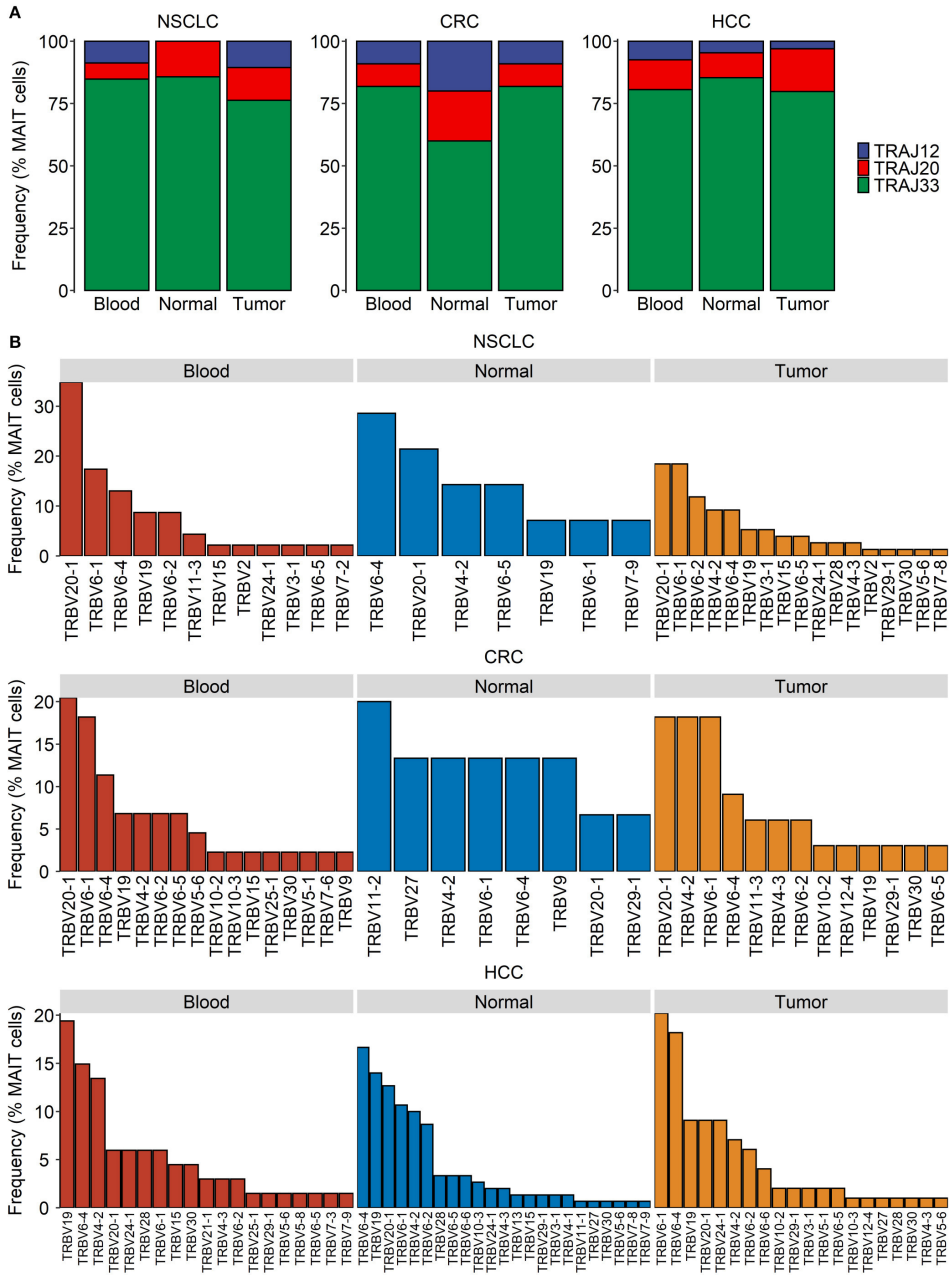
Distribution of MAIT TCR Jα and Vβ usage in cancer. Single-cell transcriptomic data were obtained for T cells from the blood, tumor, and normal tissues of patients with non-small cell lung cancer (NSCLC), colorectal cancer (CRC) and hepatocellular carcinoma (HCC). TRAJ and TRVB segment usage in MAIT cells was summarized based on assembled full-length TCR sequences. **(A,B)** MAIT cell usage of TRAJ **(A)** and TRVB **(B)** segments in indicated compartments and cancer types.

Within all the three tissue compartments examined for each of the three cancer types, TRAJ33 was the most commonly used Jα segment whereas TRAJ12 and TRAJ20 were detectable only in a minority of MAIT cells (Figure 2A). The Vβ usage of peripheral blood MAIT cells in cancer patients was heavily biased toward TRBV6 and TRBV20 (Figure 2B), consistent with previous reports on blood and hepatic MAIT cells in the absence of cancer (32). In addition, TRBV19 and TRBV4-2 were consistently detected across most tissue compartments (Figure 2B). Unexpectedly, we found TRBV19 to be the most frequently expressed Vβ segment among the peripheral blood MAIT cells of patients with HCC (Figure 2B). TRBV19 usage by blood MAIT cells has been previously reported but is not known to be common within MAIT cell TCR repertoires (78). Closer inspection of these data revealed a highly expanded MAIT cell clonotype (TRAV1-2-TRAJ33/TRBV19-TRBJ2-6) present in 18% of all MAIT cells (30/166) sampled from a solitary patient. This clone was also present in the patient's normal liver and tumor tissues, and likely accounts for the overall prevalence of TRBV19 usage observed in the HCC dataset. The predominance of TRBV6 and TRBV20 was also evident in MAIT cells from the normal and tumor tissues of the indicated cancers (Figure 2B) with the exception of the healthy colonic tissues of CRC patients, in which the usage of TRBV11-2, TRBV27, TRBV4-2, and TRBV9 was comparable to or more apparent than that of TRBV6 and TRBV20, respectively (Figure 2B). Given the low number of total cells sampled from this compartment, this finding needs to be validated in future studies.

### Intratumoral MAIT Cells Display Transcriptional Signatures of Activation and/or Exhaustion

**Figure 3:**
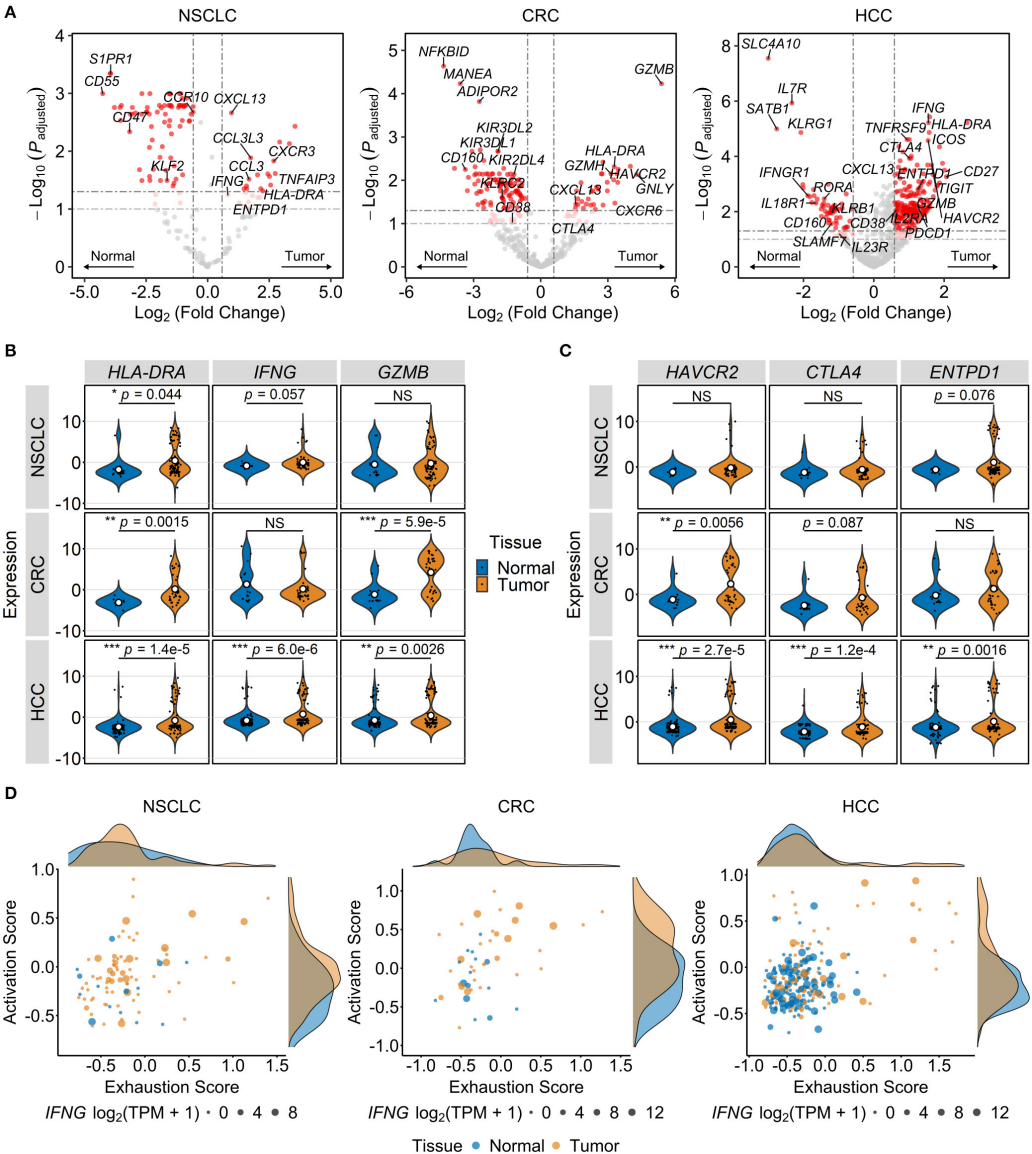
Tumor-infiltrating MAIT cells exhibit gene expression patterns consistent with activated and exhausted states. Transcriptional differences among MAIT cells of different tissue origins were analyzed in single-cell transcriptomic datasets comprising T cells from non-small cell lung cancer (NSCLC), colorectal cancer (CRC), and hepatocellular carcinoma (HCC) patients. **(A)** Volcano plots showing differentially expressed genes between MAIT cells isolated from tumors and paired unaffected tissues across indicated cancer types. Significance thresholds are *p* < 0.05 and fold-change ≥1.5. Dots are colored red if significant, light red if 0.05 ≤ *p* < 0.10, and gray if non-significant. **(B,C)** Violin plots comparing normalized expression of T cell activation/function- **(B)** and exhaustion-related genes **(C)** in tumor vs. normal tissue MAIT cells in indicated cancers. NS, non-significant. *p < 0.05, **p < 0.005, ***p < 0.001. In **(B,C)**, *p* values are calculated using Benjamini-Hochberg-adjusted limma-moderated *t*-tests. **(D)** Activation and exhaustion scores, computed by scaling followed by averaging the expression of genes in the respective lists (see Methods), are plotted for normal and tumor tissue MAIT cells. Density curves at the plot margins depict the distribution of the two scores. Dots are sized according to *IFNG* expression. TPM, transcripts per million.

To characterize changes in MAIT cell gene expression in different TMEs, we identified in each of the three cancer types DEGs between tumor-infiltrating and normal tissue MAIT cells (Figure 3A). In all three datasets, the T cell activation marker *HLA-DRA* and the exhaustion marker *CXCL13* (19, 68, 79) were upregulated in intratumoral MAIT cells (Figures 3A,B). The cytotoxic effector gene *GZMB* and the immune checkpoint gene *HAVCR2* also exhibited higher expression in MAIT cells isolated from CRC and HCC tumors (Figures 3B,C). In HCC, we observed increased expression of several more activation- (*IFNG, CD38, IL2RA, TNFRSF9*) and exhaustion-related (*TIGIT, CTLA4, PDCD1, ENTPD1*) genes in tumor MAIT cells (Figures 3A–C). Conversely, the expression of cytokine receptor-encoding genes *IL7R, IFNGR1, IL18R1*, and potentially *IL23R* (*p* = 0.065), was downregulated in HCC tumor-infiltrating MAIT cells (Figure 3A, Supplementary Table 2).

Next, we performed gene set variation analysis to identify differentially regulated pathways between tumor-infiltrating MAIT cells and those from adjacent unaffected tissues. In HCC, tumor-infiltrating MAIT cells were enriched for the GO terms “response to type I IFN” (*p* = 3.7e−8, Benjamini-Hochberg-adjusted limma-moderated *t*-test), “defense response to virus” (*p* = 8.8e−6), and “negative regulation of viral life cycle” (*p* = 4.0e−6). Consistent with the findings of our differential gene expression analysis, intratumoral MAIT cells in CRC had higher enrichment scores for “granzyme-mediated apoptotic signaling” (*p* = 0.045) and “chronic inflammatory response” (*p* = 0.045). MAIT cells within NSCLC tumors were enriched for “negative regulation of response to IFN-γ” (*p* = 0.0089) and “negative regulation of TCR signaling pathway” (*p* = 0.024), possibly reflecting the immunosuppressive effects of the NSCLC TME.

To visualize transcriptional T cell activation and exhaustion modules, we devised two cell-level signatures, one including genes that pertain to T cell activation (*CD69, CD38, HLA-DRA, IL2RA, TNFRSF9*) and function (*IFNG, TNF, GZMA, GZMB, GZMH, GNLY*) and the other consisting of immune checkpoint and dysfunction-related markers (*PDCD1, CTLA4, TIGIT, LAG3, CXCL13, ENTPD1, ITGAE, LAYN*). By scoring and plotting the two signatures across individual cells, we observed distinct tumor-specific populations of activated/exhausted MAIT cells in HCC and CRC tumors (Figure 3D). Accordingly, tumor-infiltrating MAIT cells in these cancers had significantly higher activation (*p* = 3.6e−5 and *p* = 0.01 for HCC and CRC, respectively, by Wilcoxon signed-rank test) and exhaustion scores (*p* = 0.0085 and *p* = 0.047 for HCC and CRC, respectively) when compared with adjacent normal tissue MAIT cells. There was a trend toward a higher activation score for NSCLC tumor-infiltrating MAIT cells (*p* = 0.055) while exhaustion scores for healthy tissue and intratumoral MAIT cells were similar (*p* = 0.71). As expected, in all cancer types, conventional CD8^+^ T cells had the most pronounced increase in activation and exhaustion scores within tumors relative to unaffected tissues (Supplementary Figure 1). Interestingly, activation and exhaustion scores were significantly correlated in intratumoral MAIT cells from each of the three cancer types (*p* = 6.9e−7 for HCC, *p* = 0.0065 for CRC, *p* = 9.3e−5 for NSCLC; Pearson correlation), indicating a high degree of overlap between the two signatures. When the activation-independent exhaustion program defined by Tirosh et al. (68) was assessed instead, only intratumoral MAIT cells in HCC had higher exhaustion scores than those from adjacent normal tissue (*p* = 3.0e−5 for HCC, *p* = 0.52 for CRC, *p* = 0.15 for NSCLC; Wilcoxon signed-rank test). Together, the above findings suggest that TMEs in different types of cancer perturb the transcriptional state of MAIT cells in divergent manners and to varying degrees. In HCC, intratumoral MAIT cells appear to be shifted toward an activated/exhausted phenotype whereas in CRC and NSCLC, a bias toward activation may prevail.

### PD-1 Blockade Enhances the Expression of Cytotoxic Effector Genes in Tumor-Infiltrating MAIT Cells

**Figure 4:**
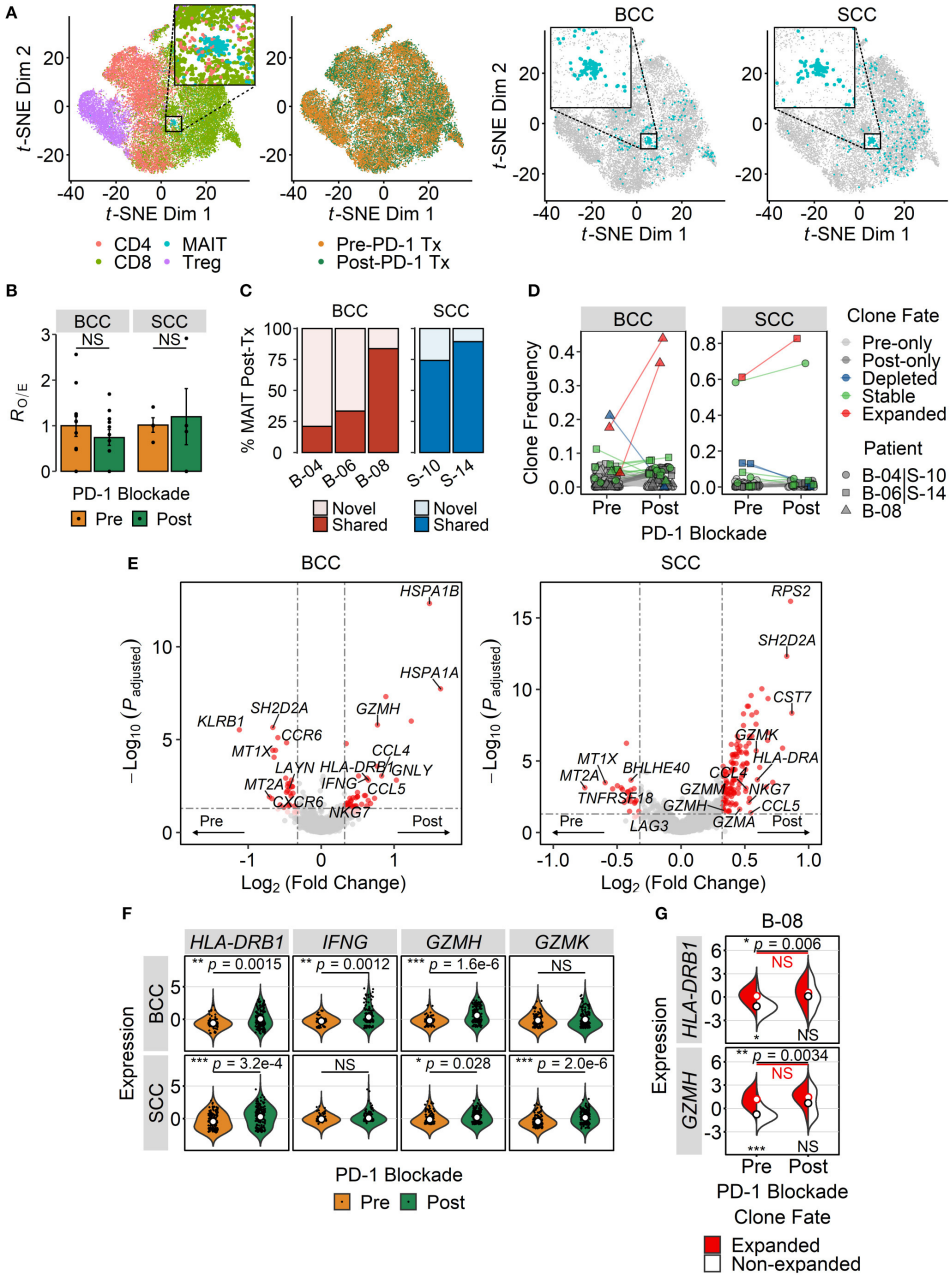
Effect of PD-1 blockade on intratumoral MAIT cell phenotype. **(A)** *t*-distributed stochastic neighbor embedding (*t*-SNE) plot of tumor-infiltrating T cells from integrated basal cell carcinoma (BCC) (*n* = 20,027) and squamous cell carcinoma (SCC) (*n* = 18,088) single-cell RNA sequencing datasets. Dots are colored according either to the T cell subset they are assigned to or to the treatment (Tx) status (first and second panels from left to right). Cells are also separately plotted for BCC and SCC (third and fourth panels from left to right), with MAIT cells shown in blue. Zoomed-in views highlight the main MAIT cell cluster. **(B)** Relative abundance of intratumoral MAIT cells before and after PD-1 blockade therapy. MAIT cell frequencies were transformed to *R*_O/E_, or the ratio of observed to expected cell number using a chi-squared test. Each dot depicts a patient (*n* = 11 for BCC, *n* = 4 for SCC), and error bars represent SEM. NS, not significant; two-sided unpaired student's *t*-test. **(C)** Clonal history of MAIT cells present in the BCC and SCC tumor microenvironments following treatment with anti-PD-1. Novel clones refer to those absent in matched pre-treatment samples, whereas shared clones are those detected both before and after PD-1 blockade. **(D)** Clonal dynamics of tumor-infiltrating MAIT cells pre- and post-treatment with anti-PD-1. Dots represent individual clones, colored according to indicated fates. Clones found exclusively in either pre- or post-treatment samples were denoted as pre- or post-only, respectively. Expanded and depleted clones were defined based on significant proportional changes by Fisher's exact test. The remaining clones were considered stable. Dot shapes correspond to different patients, assigned arbitrarily. In **(C,D)**, analysis was restricted to patients with at least 10 MAIT cells sampled from both pre- and post-treatment samples. **(E)** Volcano plots showing differentially expressed genes between MAIT cells isolated from tumors before and after PD-1 blockade. Significance thresholds are *p* < 0.05 and fold-change ≥1.25. Dots are colored red if significant, light red if 0.05 ≤ *p* ≤ 0.10, and gray if not significant. **(F)** Violin plots comparing the expression of T cell activation and effector function genes in pre- and post-treatment tumor-infiltrating MAIT cells. *P* values were calculated by Benjamini-Hochberg-adjusted limma-moderated *t*-tests. **(G)** Split-violin plots comparing gene expression in expanded and non-expanded intratumoral MAIT cell clones in a BCC patient. Open circles, colored according to their fate, represent mean values. The top pair of *p* values denote comparisons between pre- and post-treatment samples, whereas the bottom pair compare expanded and non-expanded MAIT cell clones within the same sample. *p < 0.05, **p < 0.005, ***p < 0.001; NS, not significant; Wilcoxon signed-rank test.

Having shown that MAIT cells may become activated within certain TMEs, we next explored how their transcriptional state may be influenced by immunotherapy with checkpoint inhibitors. To address this question, we took advantage of the scRNA-seq dataset from Yost et al. profiling T cells within paired BCC and SCC tumor samples pre- and post-anti-PD-1 therapy (72). MAIT cells formed distinct *t*-SNE clusters in both BCC and SCC although a few cells fell outside such clusters (Figure 4A). PD-1 blockade did not enrich or deplete MAIT cells within tumors of either cancer type (Figure 4B). We then characterized the clonal dynamics of these intratumoral MAIT cells, restricting our analysis to patients in whom at least 10 MAIT cells were detectable pre- and post-treatment to mitigate the potential effects of sampling errors. In two out of three BCC patients (B-04 and B-06), the majority of tumor-infiltrating MAIT cell clones after anti-PD-1 therapy were novel – that is they were not detected in pre-treatment samples. In contrast, in one BCC patient (B-08) and in both SCC patients, most post-treatment MAIT cell clones were already present before therapy (Figure 4C). Visualizing the fate of individual clones, we found in BCC patient B-08 that two MAIT clonotypes were significantly expanded following therapy (Figure 4D). In each of the SCC tumors, one dominant clonotype comprised the majority of intratumoral MAIT cells both before and after treatment (Figure 4D).

Differential expression analysis of pre- and post-treatment tumor-infiltrating MAIT cells revealed upregulation of the activation marker *HLA-DRB1*, the cytotoxic effector *GZMH*, and the chemokines *CCL4* and *CCL5* following anti-PD-1 therapy in both types of cancer (Figures 4E,F). In BCC, post-treatment MAIT cells also exhibited higher expression of *IFNG* and *GNLY*, both of which mediate T cell effector functions (Figures 4E,F). In SCC, several genes encoding granzymes, namely *GZMK, GZMA* and *GZMM*, exhibited significantly higher expression in intratumoral MAIT cells after PD-1 blockade (Figures 4E,F). Interestingly, in patient B-08, the two expanded MAIT cell clones had higher expression of *HLA-DRB1* and *GZMH* than the other clones at baseline, which did not further increase following PD-1 blockade (Figure 4G). In contrast, these two genes were upregulated post-treatment in non-expanded MAIT cell clones from the same patient (Figure 4G). Taken together, these results suggest that anti-PD-1 therapy promotes the expression of effector genes by MAIT cells within certain TMEs.

### Deriving a Stable and Specific MAIT Cell Gene Signature

**Figure 5:**
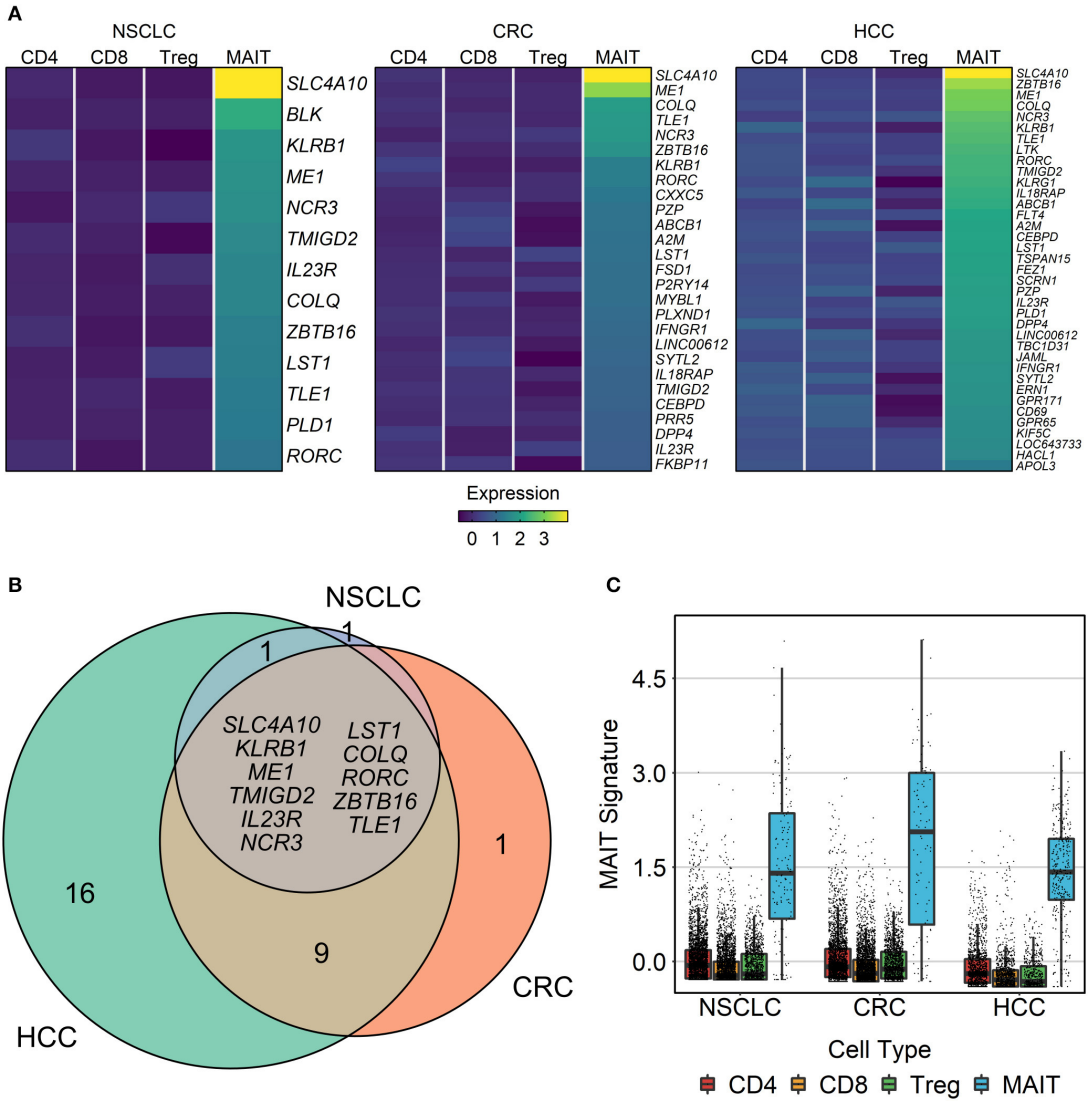
Identification of MAIT cell signature genes. Three single-cell transcriptomic datasets comprising T cells from the blood, tumor, and adjacent healthy tissue of patients with non-small cell lung cancer (NSCLC), colorectal cancer (CRC), and hepatocellular carcinoma (HCC) were analyzed. Within each compartment of each cancer type, genes with significantly higher expression in MAIT cells than in other T cell subsets (conventional CD4^+^, conventional CD8^+^, and regulatory T cells) were identified using limma-trend (Benjamini-Hochberg-adjusted *t*-test yielding *p* < 0.1). **(A)** Heatmap showing the mean normalized expression of MAIT cell markers across T cell subsets in each cancer. **(B)** Venn diagram of overlapping MAIT cell marker genes between cancer types. Listed genes comprise the eleven-gene MAIT cell signature that remained stable across cancer contexts. **(C)** Validation of the MAIT cell signature. Expression of the genes listed in **(B)** was scaled, averaged, and then plotted for each cell. Boxes extend between quartiles, and whiskers extend to ±1.5× interquartile range.

Using the HCC, CRC and NSCLC scRNA-seq datasets, we next sought to define a set of marker genes that consistently distinguish MAIT cells from other T cell populations across various tissue compartments and cancer types. To this end, we identified genes with significantly higher expression in MAIT cells than in non-MAIT T cells (divided into CD8, CD4, and Treg subsets) (Figure 5A, Supplementary Table 3). Within all three cancer types, a set of genes could be defined that remained stably overexpressed in MAIT cells regardless of their tissue/site origin (Supplementary Figure 2). The intersection of these three cancer-specific gene sets, comprising eleven genes (*SLC4A10, KLRB1, ME1, TMIGD2, IL23R, NCR3, LST1, COLQ, RORC, ZBTB16, TLE1*) that represent the most robust MAIT cell markers, was used for downstream analyses in bulk tumor transcriptomic datasets (Figure 5B). We then collapsed the expression of these marker genes into a single index, the “MAIT cell signature,” and demonstrated the specific enrichment of this signature in MAIT cells relative to non-MAIT T cell subsets (Figure 5C). Crucially, our consensus-based approach for deriving a MAIT cell signature selects for generalizability of markers across tissue contexts.

### Validating the MAIT Signature in Additional Transcriptomic Datasets

To further validate the specificity and stability of the MAIT cell signature, we tested its performance in several independent gene expression datasets. A recent study by Gutierrez et al. generated bulk and single-cell transcriptomic data of innate-like T cells isolated from the peripheral blood, including MAIT, *i*NKT, Vδ1, and Vδ2 T cells (69). The authors also profiled conventional CD4^+^ and CD8^+^ T cells as well as NK cells for comparison purposes. We first examined bulk RNA-seq data from the purified lymphocyte populations. We found the expression of most MAIT signature genes to be highly specific to MAIT cells even in comparison with other innate-like subsets like *i*NKT cells, which are known to share a similar transcriptional profile (Supplementary Figure 3A) (51, 76). Out of 66 pairwise comparisons (11 genes × 6 cell types), all but 8 pointed to significantly elevated levels in MAIT cells (*p* < 0.05, limma-moderated *t*-tests). Four genes were expressed at comparable levels in *i*NKT cells (*KLRB1, COLQ, ZBTB16, TLE1*), three in NK cells (*TMIGD2, COLQ, ZBTB16*), and one in Vδ2 cells (*TLE1*) (Supplementary Figure 3A). As expected, the aggregate MAIT signature score was significantly enriched in MAIT cells relative to every other lymphocyte population (*p* < 0.0001, by Wilcoxon signed-rank test) (Supplementary Figure 3B). Results from the scRNA-seq dataset were concordant, with all MAIT signature genes being highly expressed in MAIT cells, and a few expressed at similar levels by *i*NKT, Vδ2, and NK cells (Supplementary Figure 3C). The performance of the MAIT signature was also verified on a per-cell basis (largest *p* = 1.3e−13, compared to *i*NKT cells) (Supplementary Figure 3D).

Since we derived the MAIT signature from transcriptomic profiles of tumor-infiltrating T cells, its constituent genes were selected based on their discriminatory capacity relative to other T cell subsets, but not necessarily non-T cell populations. Therefore, we next evaluated this signature in more complex, unpurified cell mixtures better representing the crude immune compartment of TMEs. To this end, we re-processed and analyzed scRNA-seq data from primary tumors of HCC patients, generated by Ma et al. (70), which contained diverse malignant, stromal and immune cell populations (Supplementary Figures 4A,B). While there was no paired scTCR-seq data with which to identify MAIT cells in this case, we could still ask whether each MAIT signature gene tended to be expressed specifically among T cells, or more promiscuously across other cell types. Of the 11 genes comprising the MAIT signature, five were expressed primarily by T cells (*SLC4A10, KLRB1, TMIGD2, IL23R, NCR3*), five had higher expression among non-T cell types (*ME1, LST1, RORC, ZBTB16, TLE1*), and one (*COLQ*) was nearly undetectable across all cells (Supplementary Figure 4C). Removing the five genes with high expression in non-T cell types from the MAIT signature improved its specific enrichment among T cells, although we cannot ascertain whether T cells with high MAIT signature scores represent bona fide MAIT cells (Supplementary Figure 4D). Hierarchical clustering of the MAIT signature genes alongside pan-T cell markers (*CD3D, CD3E, CD3G*) divided these into two groups, one consisting of the genes whose expression tended to be positively associated with the *CD3* genes and with each other (*NCR3, KLRB1, TMIGD2, IL23R, LST1, SLC4A10, COLQ*), and the other comprising the genes whose expression was generally anticorrelated with the *CD3* genes (*ZBTB16, TLE1, ME1, RORC*) (Supplementary Figure 4E). Notably, the latter group largely overlapped with the genes whose expression was found to be nonspecific to T cells. While the degree of specificity for each MAIT cell signature gene will likely vary between tissue compartments, disease states, individuals, and methods of measurement, these results suggest that the MAIT signature may be amenable to refinement in-context based on expected patterns of correlated gene expression.

Using the datasets from Yost et al., the MAIT signature performed well in BCC and SCC tumors both before and after anti-PD-1 therapy, suggesting that it is reasonably robust to changes in biological state (Supplementary Figures 5A,B). Interestingly, MAIT cells in BCC tumors post-treatment had significantly decreased MAIT signature scores (*p* < 0.0001, by Wilcoxon signed-rank test) (Supplementary Figure 5A), while the same was not true for SCC tumors (Supplementary Figure 5B). This finding lends further support to the notion that PD-1 blockade can alter the transcriptional profile of intratumoral MAIT cells, a finding that needs to be validated in larger cohorts and linked to markers of response to therapy. Despite this effect, the signature was still specifically enhanced in MAIT cells compared to all other T cell subsets in post-treatment BCC samples (largest *p* < 0.005, compared to CD4^+^ T cells).

To investigate whether our MAIT signature may be useful in estimating MAIT cell frequencies in bulk RNA-seq data, we generated simulated pseudo-bulk samples from these scRNA-seq profiles. In the BCC dataset, we found the MAIT signature score to strongly correlate with the frequency of MAIT cells, but not that of conventional CD8^+^ T cells, CD4^+^ T cells, or Tregs (Supplementary Figure 5C). Moreover, this correlation was reproducible, regardless of pre- or post-treatment sampling, for both BCC and SCC (Supplementary Figures 5D,E). In line with the observed perturbation of the MAIT signature by PD-1 blockade in BCC, the correlation between the MAIT signature score and MAIT cell frequency was weaker when restricting simulations to anti-PD-1-treated tumors (Supplementary Figure 5D). These results, supplemented by our other means of validation, demonstrate the value of the MAIT signature in estimating MAIT cell frequencies within bulk gene expression datasets.

### Refining the MAIT Signature Using Bulk Tumor Transcriptomic Data

TCGA provides matched clinical and bulk tumor RNA-seq data across many human cancers, which enabled us to evaluate the prognostic significance of the above-defined MAIT signature. We obtained data from 20 TCGA cancers, focusing mainly on solid tumors with relatively large sample sizes (Supplementary Table 1). As an additional quality control step before testing for associations with clinical outcomes, we evaluated the performance of our MAIT signature using gene expression data from these 20 cancers.

Most genes included in the MAIT signature showed significant positive correlations with the expression of the pan-T cell genes *CD3D, CD3E* and *CD3G*, which was expected given that MAIT cell abundance within tumors, estimated in absolute terms by our signature and its constituent genes, likely scales broadly with the extent of overall T cell infiltration (Supplementary Figure 6). We also observed positive correlations between the expression of MAIT signature genes and seven curated NK cell markers (80, 81), which may reflect the promiscuous expression of MAIT marker genes by NK cells, of NK marker genes by T cells (thus returning to the previous point), or both (Supplementary Figure 6). Of note, in every cancer type, there were MAIT signature genes whose expression did not correlate with that of pan-T cell markers or other MAIT signature genes. As shown when evaluating the MAIT signature in scRNA-seq data profiling the whole HCC TME, this “missing” correlation for certain MAIT signature genes in bulk transcriptomic data may be due to their expression in non-T cell populations within TMEs, which then confounds and overpowers the underlying correlation in abundance between the transcripts of MAIT origin. Indeed, RNA-seq data in purified leukocyte populations from the Human Blood Atlas revealed that *TLE1* and *LST1* are highly expressed in other immune cell subsets such as monocytes and basophils (Supplementary Figure 7). Similar reasoning also holds for tumor and stromal cells. Therefore, we devised rules to define “blocks” of correlation (see Methods for details), whereby the gene set comprising the MAIT signature for each cancer is independently trimmed until only genes that exhibit significant positive correlations, where expected, remain. Using this approach, we found that four cancers, namely urothelial carcinoma, cervical squamous cell carcinoma, low-grade glioma and glioblastoma, did not display a robust transcriptional signal of MAIT cell infiltration, as fewer than five of the eleven original MAIT signature genes were kept (Supplementary Figure 6B). These cancers were thus excluded from downstream analyses. We then scored the degree of MAIT cell infiltration in each tumor sample by normalizing and averaging the expression of the MAIT cell signature genes selected for each cancer type. A final adjustment was made to the MAIT cell score by regressing out the T cell score, similarly computed based on the expression of *CD3D, CD3E* and *CD3G*, thereby normalizing the MAIT cell abundance estimate in each sample against the degree of total T cell infiltration.

### Association of the MAIT Signature With Clinical Outcomes Across Human Cancers

**Figure 6:**
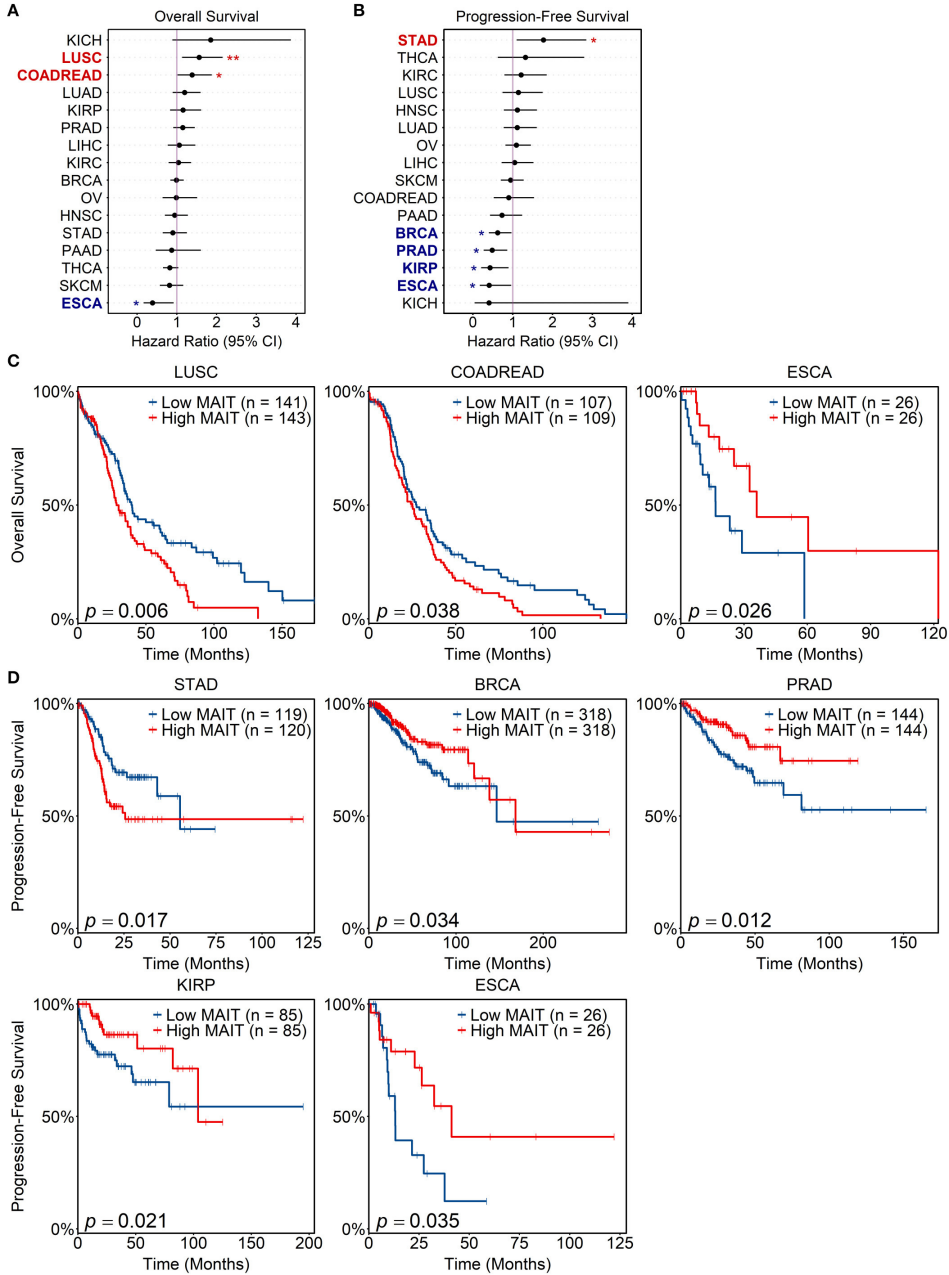
Prognostic value of MAIT cell signature in human cancers. Matched tumor transcriptomic and clinical data for 16 cancers in The Cancer Genome Atlas (TCGA) were accessed and analyzed. For each sample, a MAIT cell score was computed, which estimates the abundance of MAIT cells within the tumor, normalized for overall T cell infiltration (see Methods). Univariate cox proportional hazard models were tested for each cancer using the MAIT score as a predictor variable (patients stratified at 33rd and 67th percentiles) and either overall or progression-free survival as the endpoint. **(A,B)** Forest plots of hazard ratios for overall **(A)** and progression-free survival **(B)** across TCGA cancers. Cancer types with significant survival associations are bolded and shown in red if a MAIT score is negatively prognostic, or blue if it is positively prognostic. Lines span 95% confidence intervals. *p < 0.05, **p < 0.01. **(C,D)** Kaplan-Meier curves visualizing significant associations between MAIT scores and overall **(C)** and progression-free **(D)** survival. Log-rank tests were employed to compare curves. Cancer type abbreviations are expanded in Supplementary Table 1.

To explore potential links between intratumoral MAIT cell abundance and patient survival across human cancers, we tested a series of Cox proportional hazard models using the MAIT cell score to stratify patients into MAIT-high and MAIT-low groups for each cancer type. We found that a high MAIT score was associated with improved overall survival (OS) in EAC, but poorer OS in CRC and lung squamous cell carcinoma (Figures 6A,C). When considering progression-free survival (PFS), a high MAIT score was associated with favorable prognoses in breast invasive carcinoma, prostate adenocarcinoma, papillary renal cell carcinoma and EAC, while it portended earlier progression in stomach adenocarcinoma (Figures 6B,D). Notably, in HCC, where intratumoral MAIT cell abundance has been linked to improved or poor survival in seemingly contradictory reports (19, 20), we did not find any prognostic associations between the MAIT score and either OS or PFS [Figures 6A,B; shown using the TCGA abbreviation for liver hepatocellular carcinoma (LIHC)]. Overall, these results indicate that the prognostic value of MAIT cell infiltration into tumors varies by cancer type and endpoint definition, reinforcing the need for future studies that independently and systematically test for such associations across a wide spectrum of human malignancies.

One potential determinant of MAIT cell abundance in TMEs is the expression of *MR1*, the gene encoding the antigen-presenting molecule involved in cognate MAIT cell activation. We found positive correlations between *MR1* expression and MAIT scores in CRC (*p* = 0.0081, Pearson correlation), stomach adenocarcinoma (*p* = 0.0015), prostate adenocarcinoma (*p* = 1.29e−10), lung adenocarcinoma (*p* = 0.00088), and clear cell renal cell carcinoma (*p* = 0.013). The expression of *MR1* was not significantly associated with OS or PFS in any of the above cancers but showed trending *p* values for worse OS in CRC (*p* = 0.078 by log-rank test), worse PFS in stomach adenocarcinoma (*p* = 0.081), and better PFS in prostate adenocarcinoma (*p* = 0.10). These trends are in the same directions as the prognostic links identified for MAIT scores, which warrants further investigation.

## Discussion

In this study, we present the first *in silico* cross-cancer analysis of MAIT cells using available bulk and single-cell transcriptomic datasets. Our analyses reproduced several established findings about MAIT cells in the context of human malignancies. In the HCC scRNA-seq dataset, we found that MAIT cells were reduced within tumors compared to the adjacent normal tissue and enriched in the healthy liver compared to peripheral blood, consistent with previous studies (19, 20, 42). We did not, however, replicate the finding that MAIT cells are significantly enriched in CRC tumors compared to their surrounding unaffected tissue, which has been reported in a number of independent studies (16, 17, 26). The CRC dataset included 9,878 T cells from 11 patients in total, which might have made our analysis underpowered.

Our examination of the TCR repertoire of MAIT cells validated their preferential usage of TRAJ33 in the blood, normal tissues and tumors across the three cancer types. In contrast, the Vβ bias we observed was more flexible and context-dependent. Previous studies in individuals without cancer have established the preferential usage of TRBV6 and TRBV20 by peripheral blood and hepatic MAIT cells (32), which is generally consistent with our present findings across the tissue compartments examined. Surprisingly, however, MAIT cells sampled from the healthy colonic tissue of CRC patients expressed TRBV11-2, TRBV27, TRBV4-2, TRBV9, and TRBV6 at similar frequencies. While this observation was based on a relatively small sample, there is reason to speculate that malignancy and the tissue context might reshape the TCR repertoire of MAIT cells. The TCRβ chain expressed by MAIT cells has been shown to influence their MR1-dependent responses to microbial antigens (78, 82). It is therefore possible that tissue-resident commensal bacteria in the gut, for instance, may cause differential expansion of MAIT cell clones depending on the TCRβ chain utilized, which may confer varying avidities for cognate stimuli. Moreover, given the complex interplay between cancer and tissue dysbiosis (83, 84), the TCR composition of MAIT cells in cancer patients may exhibit different biases than that in healthy individuals. Further studies are needed to compare and contrast the TCR repertoire of tissue-resident or even passenger MAIT cells in normal and pathological states.

In agreement with previous work, we show that MAIT cells in HCC tumors express higher levels of T cell activation (*CD38, HLA-DRA*) and exhaustion markers (*PDCD1, CTLA4, HAVCR2*), but lower levels of the effector function-associated genes *CD160* and *KLRG1* (Figure 3A) (20). We extended this finding by demonstrating that intratumoral MAIT cells in HCC also exhibit higher expression of the effector genes *GZMB* and *IFNG*, even though studies of HCC and colorectal liver metastases have shown that MAIT cells isolated from tumor masses are suppressed in terms of T_H_1 cytokine secretion and granzyme B production when stimulated *ex vivo* (18, 20). This hypofunctional state may be explained in part by downregulation of cytokine receptors (*IL7R, IL18R1, IFNGR1, IL23R*) that mediate MAIT cell activation and modulate their downstream effector programs (Figure 3A, Supplementary Table 2) (42, 44, 45). Functional impairments in CRC have also been reported (26), and our present work indicates the upregulation of several T cell activation- and exhaustion-related genes in CRC-infiltrating MAIT cells (Figure 3D). The elevated expression of *GZMB* in MAIT cells from HCC and CRC tumors raises the possibility that the cytotoxic activity of these cells may have been bolstered by the respective TMEs. In support of this notion, Sundström et al. showed that colon tumor-infiltrating MAIT cells and their healthy tissue counterparts retain a comparable cytotoxic potential when activated *ex vivo* (85).

Our group has previously described the simultaneous expression of activation and exhaustion programs in MAIT cells in conjunction with heavy-handed cytokine responses to bacterial superantigens (86). Stimulation of MAIT cells with staphylococcal enterotoxin B, a potent superantigen, causes their hyperactivation together with upregulation of the co-inhibitory markers LAG-3 and TIM-3 (*HAVCR2*) and anergy as characterized by impaired cytokine production upon secondary challenge with bacterial lysates, which could be reversed by blocking LAG-3 with a monoclonal antibody (86). Taken together, these observations further the rationale for attempting to restore the pro-inflammatory and cytotoxic functionality of tumor-infiltrating MAIT cells with immune checkpoint inhibitors. While a skewed MAIT cell response toward IL-17 production has been proposed to potentially promote tumor progression (21, 87, 88), *IL17A* transcripts were undetectable in most MAIT cells within the datasets analyzed, possibly reflecting their relatively low baseline abundance. More work is needed to directly characterize the effector functions of intratumoral MAIT cells *in situ*, which is likely dictated, in conjunction with their transcriptional state, by the coordinated action of local stimulatory and inhibitory cues.

Our survival analyses independently replicated the observation that MAIT cell infiltration into CRC tumors is a negative prognostic factor for OS (17). Although we showed a high MAIT cell score to be associated with both improved OS and PFS in EAC, a previous study on this cancer did not detect a correlation between the intratumoral MAIT cell frequency and overall patient survival (21). Interestingly, however, they did find that MAIT cells were more abundant in EAC tumors without nodal involvement, which have a more favorable prognosis (21). The associations we identified in other cancer types between the MAIT score and patient survival may help guide the prioritization of MAIT cell studies in human malignancies but will need to be validated in additional cohorts using traditional immunological methods to measure MAIT cell abundance within tumors. Seemingly contradictory results arising from two independent studies on the prognostic significance of HCC-infiltrating MAIT cells may have stemmed, at least in part, from the technical approaches employed (19, 20). Zheng et al. utilized the TCGA gene expression data (19) whereas Duan et al. resorted to flow cytometry, quantitative polymerase chain reaction and immunohistochemistry (20) to reach their respective conclusions. Another difference between these two studies is the rate of hepatitis B virus (HBV) infection among HCC patients, which was low in the TCGA cohort (89) and highly prevalent in the cohorts studied by Duan et al. (20). Chronic HBV infection is the most common risk factor for HCC (90), and has recently been associated with peripheral blood MAIT cell activation and exhaustion although its impact on hepatic MAIT cells is less clear (91–93). Our pathway enrichment analysis in the HCC scRNA-seq dataset generated by Zheng et al., in which all patients were HBV-positive unlike in the TCGA cohort, revealed the upregulation of antiviral gene modules in tumor-infiltrating MAIT cells. Therefore, the phenotypic and functional attributes of intratumoral MAIT cells may be influenced by comorbid viral infections. These considerations reinforce the need to test and re-test exploratory hypotheses in primary cohorts with comprehensive clinical records. In our survival analyses, we did not detect any associations between our MAIT signature and either OS or PFS in HCC (Figures 6A,B).

As the three cancer scRNA-seq datasets we analyzed were generated to profile the entire T cell compartment rather than MAIT cells specifically, some of our approaches were likely not fully powered to discern subtle effects and differences. In order to more finely characterize the clonal dynamics and tissue-mediated transcriptional changes of MAIT cells in cancer, future studies will need to prioritize cell sampling and data collection strategies accordingly. In particular, approaches to identify distinct MAIT cell subsets or to infer the developmental trajectory of such clusters will likely require datasets focused exclusively on this population.

The MAIT signature and constituent markers that we derived herein can be applied to extract additional information from existing and future transcriptomic datasets. The availability of reconstructed TCR sequences in the datasets we analyzed enabled us to define MAIT cells by their invariant TCRα chain. However, the gene signature we defined in this work should help detect MAIT cells when single-cell TCR sequencing data are not available. This approach may need to be further optimized or validated using additional biological measures and sequencing platforms. Other than this gene signature, MAIT cell abundance can be estimated in bulk gene expression data using algorithms that extract TCR transcripts from RNA-seq reads (94, 95). This approach has a much higher specificity, though likely at the cost of sensitivity, and would allow the validation of survival associations identified in this study.

In summary, by analyzing public transcriptomic datasets, we demonstrate that MAIT cells bear some shared clonotypes in the blood, normal tissues and tumors of cancer patients. MAIT cells show evidence of both activation and exhaustion within some but not all TMEs. Importantly, the intratumoral abundance of MAIT cells is associated with patient outcomes in several human malignancies. Based on our analyses, we suggest that MAIT cells play important roles within TMEs where they engage in cross-talk with other players, resulting in their activation and/or exhaustion. Finally, we provide a resource for MAIT cell-focused *in silico* analyses of high-dimensional cancer omics data.

## Data Availability Statement

All datasets presented in this study are included in the article/Supplementary Material.

## Author Contributions

TY participated in study design and data interpretation, collected and analyzed data, and wrote the manuscript. PS assisted with data collection and analysis. SMMH conceived and supervised the project, participated in study design and data interpretation, and edited the manuscript. All authors contributed to the article and approved the submitted version.

## Conflict of Interest

The authors declare that the research was conducted in the absence of any commercial or financial relationships that could be construed as a potential conflict of interest.
