# Supplementary material for: Leveraging Public Single-Cell and Bulk Transcriptomic Datasets to Delineate MAIT Cell Roles and Phenotypic Characteristics in Human Malignancies
Source: Front Immunol. 2020 Jul 31;11:1691. doi: 10.3389/fimmu.2020.01691 (PMC7413026; doi:10.3389/fimmu.2020.01691)
Supplement: Supplementary file 2 [file Presentation_1.PPTX]

## Slide 1
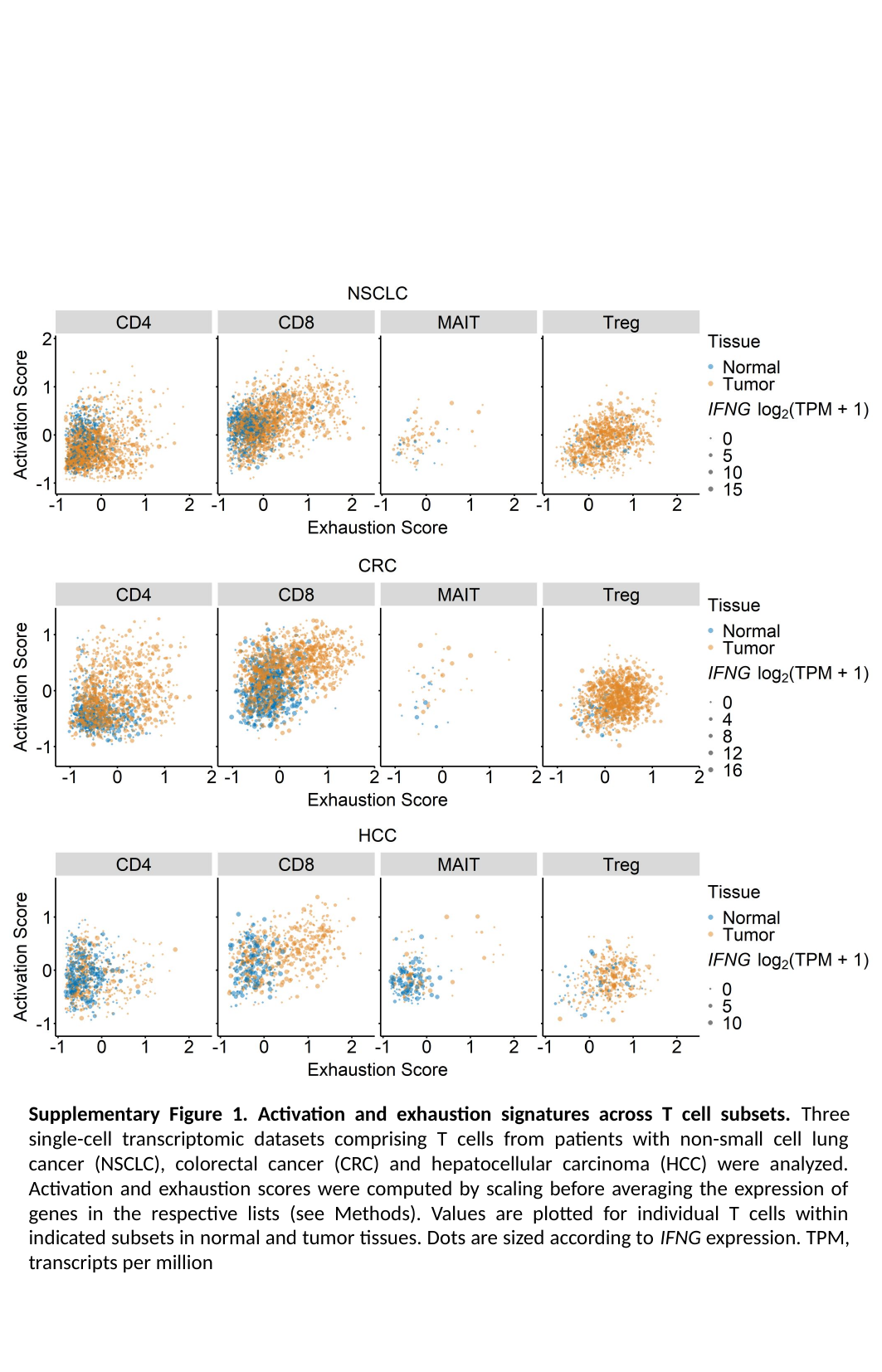

Supplementary Figure 1. Activation and exhaustion signatures across T cell subsets. Three single-cell transcriptomic datasets comprising T cells from patients with non-small cell lung cancer (NSCLC), colorectal cancer (CRC) and hepatocellular carcinoma (HCC) were analyzed. Activation and exhaustion scores were computed by scaling before averaging the expression of genes in the respective lists (see Methods). Values are plotted for individual T cells within indicated subsets in normal and tumor tissues. Dots are sized according to IFNG expression. TPM, transcripts per million

## Slide 2
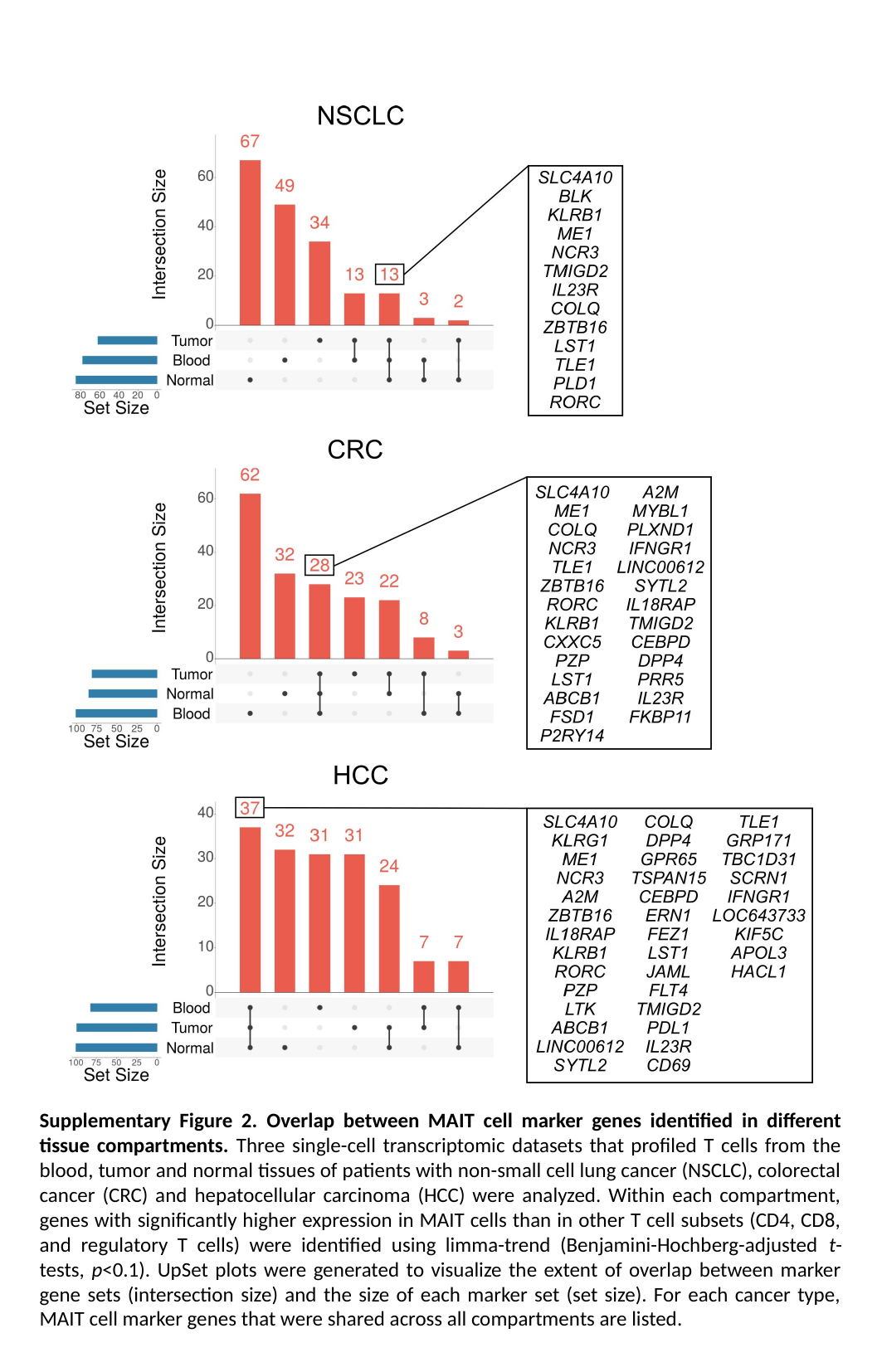

Supplementary Figure 2. Overlap between MAIT cell marker genes identified in different tissue compartments. Three single-cell transcriptomic datasets that profiled T cells from the blood, tumor and normal tissues of patients with non-small cell lung cancer (NSCLC), colorectal cancer (CRC) and hepatocellular carcinoma (HCC) were analyzed. Within each compartment, genes with significantly higher expression in MAIT cells than in other T cell subsets (CD4, CD8, and regulatory T cells) were identified using limma-trend (Benjamini-Hochberg-adjusted t-tests, p<0.1). UpSet plots were generated to visualize the extent of overlap between marker gene sets (intersection size) and the size of each marker set (set size). For each cancer type, MAIT cell marker genes that were shared across all compartments are listed.

## Slide 3
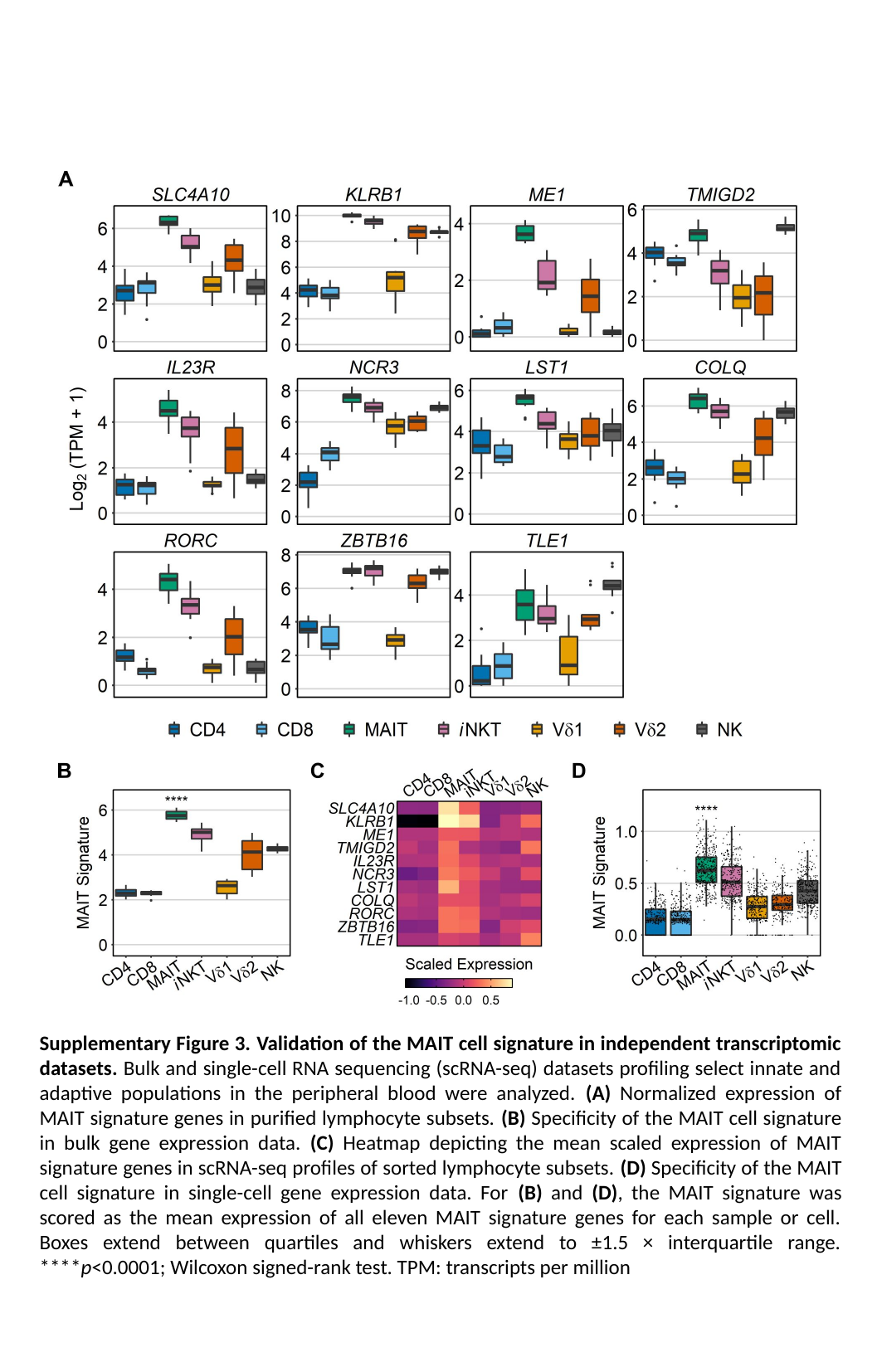

Supplementary Figure 3. Validation of the MAIT cell signature in independent transcriptomic datasets. Bulk and single-cell RNA sequencing (scRNA-seq) datasets profiling select innate and adaptive populations in the peripheral blood were analyzed. (A) Normalized expression of MAIT signature genes in purified lymphocyte subsets. (B) Specificity of the MAIT cell signature in bulk gene expression data. (C) Heatmap depicting the mean scaled expression of MAIT signature genes in scRNA-seq profiles of sorted lymphocyte subsets. (D) Specificity of the MAIT cell signature in single-cell gene expression data. For (B) and (D), the MAIT signature was scored as the mean expression of all eleven MAIT signature genes for each sample or cell. Boxes extend between quartiles and whiskers extend to ±1.5 × interquartile range. ****p<0.0001; Wilcoxon signed-rank test. TPM: transcripts per million

## Slide 4
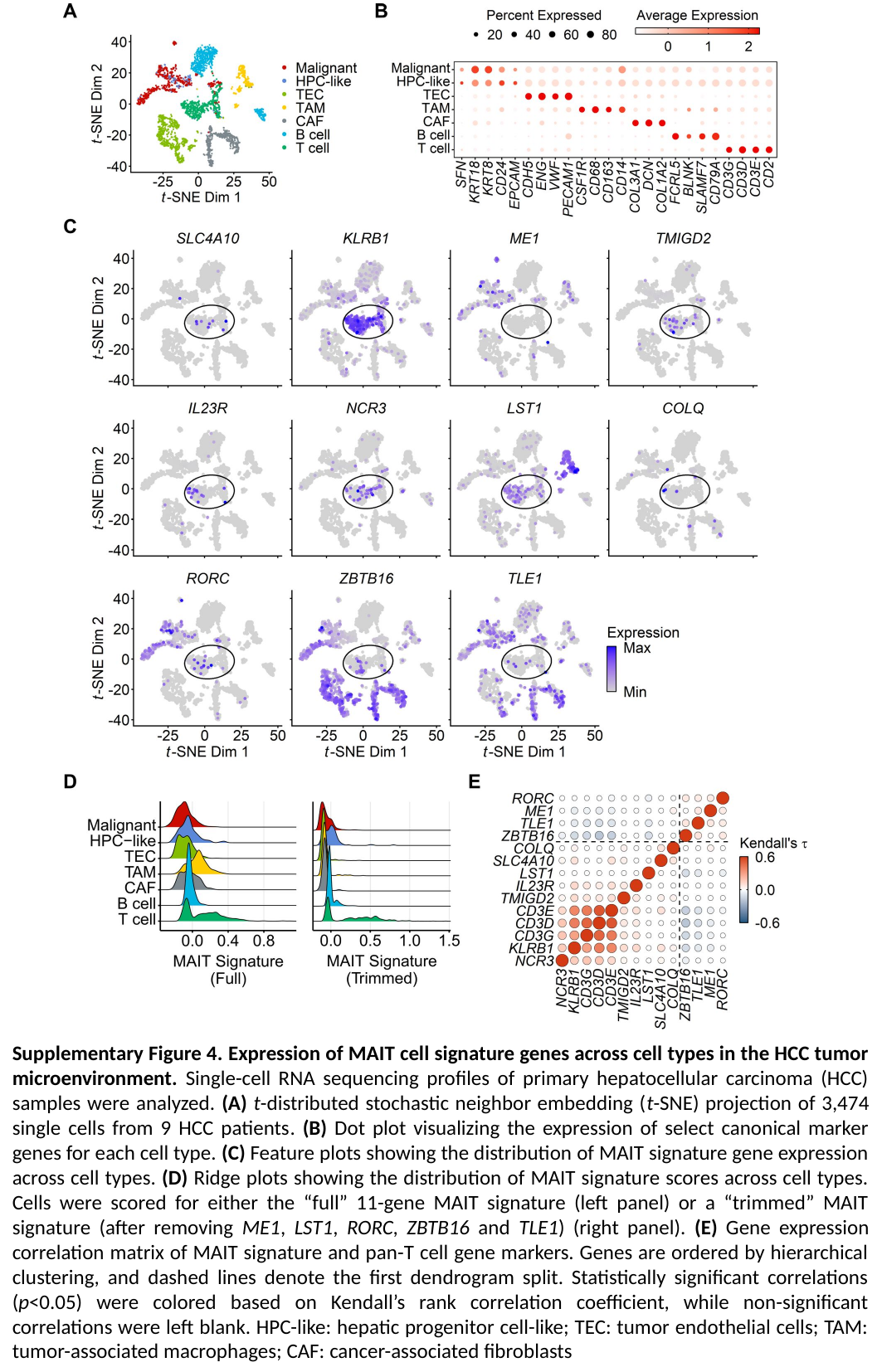

Supplementary Figure 4. Expression of MAIT cell signature genes across cell types in the HCC tumor microenvironment. Single-cell RNA sequencing profiles of primary hepatocellular carcinoma (HCC) samples were analyzed. (A) t-distributed stochastic neighbor embedding (t-SNE) projection of 3,474 single cells from 9 HCC patients. (B) Dot plot visualizing the expression of select canonical marker genes for each cell type. (C) Feature plots showing the distribution of MAIT signature gene expression across cell types. (D) Ridge plots showing the distribution of MAIT signature scores across cell types. Cells were scored for either the “full” 11-gene MAIT signature (left panel) or a “trimmed” MAIT signature (after removing ME1, LST1, RORC, ZBTB16 and TLE1) (right panel). (E) Gene expression correlation matrix of MAIT signature and pan-T cell gene markers. Genes are ordered by hierarchical clustering, and dashed lines denote the first dendrogram split. Statistically significant correlations (p<0.05) were colored based on Kendall’s rank correlation coefficient, while non-significant correlations were left blank. HPC-like: hepatic progenitor cell-like; TEC: tumor endothelial cells; TAM: tumor-associated macrophages; CAF: cancer-associated fibroblasts

## Slide 5
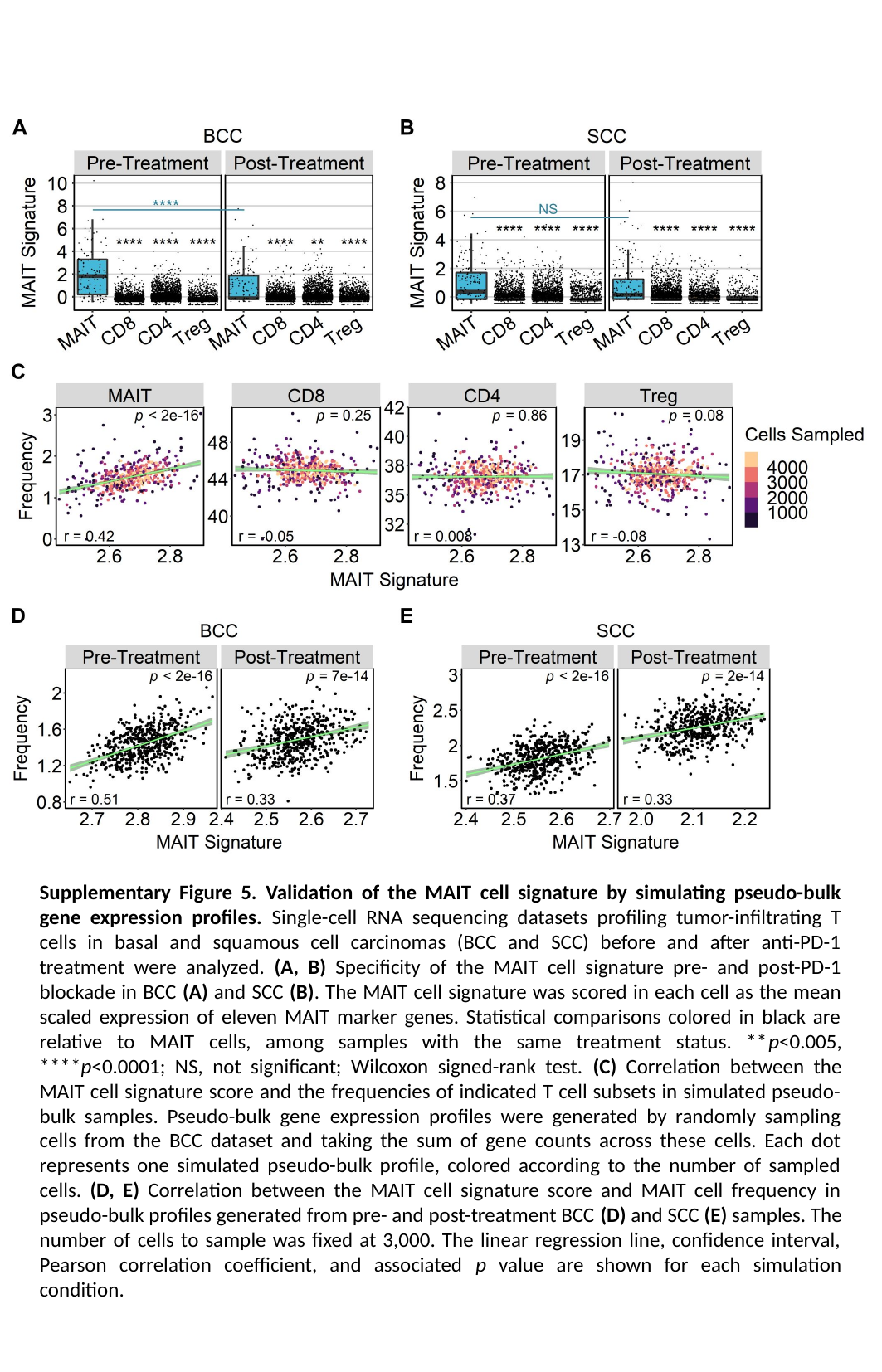

Supplementary Figure 5. Validation of the MAIT cell signature by simulating pseudo-bulk gene expression profiles. Single-cell RNA sequencing datasets profiling tumor-infiltrating T cells in basal and squamous cell carcinomas (BCC and SCC) before and after anti-PD-1 treatment were analyzed. (A, B) Specificity of the MAIT cell signature pre- and post-PD-1 blockade in BCC (A) and SCC (B). The MAIT cell signature was scored in each cell as the mean scaled expression of eleven MAIT marker genes. Statistical comparisons colored in black are relative to MAIT cells, among samples with the same treatment status. **p<0.005, ****p<0.0001; NS, not significant; Wilcoxon signed-rank test. (C) Correlation between the MAIT cell signature score and the frequencies of indicated T cell subsets in simulated pseudo-bulk samples. Pseudo-bulk gene expression profiles were generated by randomly sampling cells from the BCC dataset and taking the sum of gene counts across these cells. Each dot represents one simulated pseudo-bulk profile, colored according to the number of sampled cells. (D, E) Correlation between the MAIT cell signature score and MAIT cell frequency in pseudo-bulk profiles generated from pre- and post-treatment BCC (D) and SCC (E) samples. The number of cells to sample was fixed at 3,000. The linear regression line, confidence interval, Pearson correlation coefficient, and associated p value are shown for each simulation condition.

## Slide 6
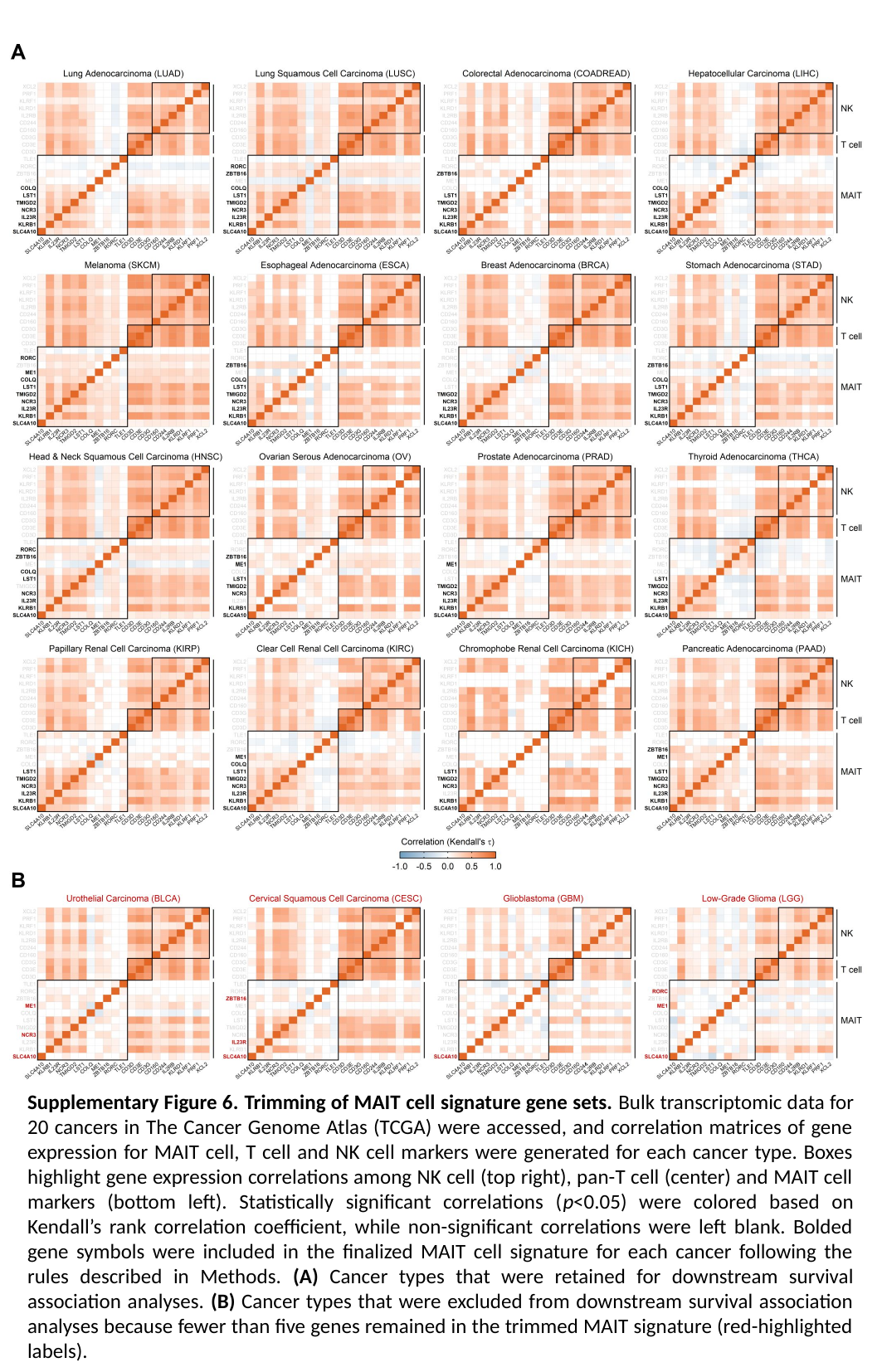

Supplementary Figure 6. Trimming of MAIT cell signature gene sets. Bulk transcriptomic data for 20 cancers in The Cancer Genome Atlas (TCGA) were accessed, and correlation matrices of gene expression for MAIT cell, T cell and NK cell markers were generated for each cancer type. Boxes highlight gene expression correlations among NK cell (top right), pan-T cell (center) and MAIT cell markers (bottom left). Statistically significant correlations (p<0.05) were colored based on Kendall’s rank correlation coefficient, while non-significant correlations were left blank. Bolded gene symbols were included in the finalized MAIT cell signature for each cancer following the rules described in Methods. (A) Cancer types that were retained for downstream survival association analyses. (B) Cancer types that were excluded from downstream survival association analyses because fewer than five genes remained in the trimmed MAIT signature (red-highlighted labels).

## Slide 7
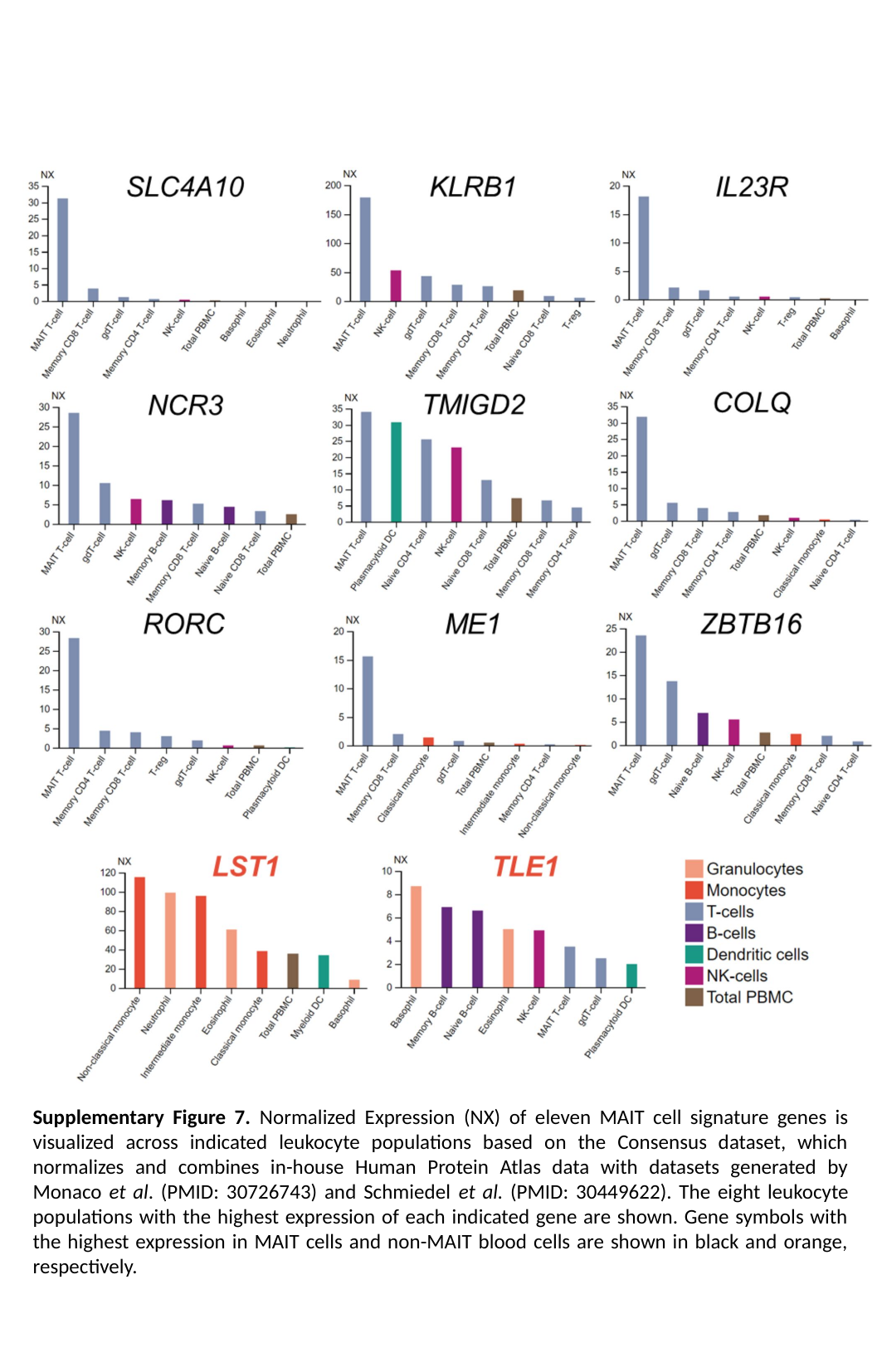

Supplementary Figure 7. Normalized Expression (NX) of eleven MAIT cell signature genes is visualized across indicated leukocyte populations based on the Consensus dataset, which normalizes and combines in-house Human Protein Atlas data with datasets generated by Monaco et al. (PMID: 30726743) and Schmiedel et al. (PMID: 30449622). The eight leukocyte populations with the highest expression of each indicated gene are shown. Gene symbols with the highest expression in MAIT cells and non-MAIT blood cells are shown in black and orange, respectively.
